# Heat shock proteins (HSPs) in non-alcoholic fatty liver disease (NAFLD): from molecular mechanisms to therapeutic avenues

**DOI:** 10.1186/s40364-024-00664-z

**Published:** 2024-10-12

**Authors:** Zhenwang Nie, Congshu Xiao, Yingzi Wang, Rongkuan Li, Fangcheng Zhao

**Affiliations:** 1 Infectious Disease Department, The Second Hospital of Dalian Medical University, Dalian, China; 2 International Medical Department, The Second Hospital of Dalian Medical University, Dalian, China

**Keywords:** HSP60, HSP70, HSP90, GRP78, NAFLD, Molecular mechanisms, Therapeutic strategies.

## Abstract

Non-alcoholic fatty liver disease (NAFLD), a spectrum of liver conditions characterized by fat accumulation without excessive alcohol consumption, represents a significant global health burden. The intricate molecular landscape underlying NAFLD pathogenesis involves lipid handling, inflammation, oxidative stress, and mitochondrial dysfunction, with endoplasmic reticulum (ER) stress emerging as a key contributor. ER stress triggers the unfolded protein response (UPR), impacting hepatic steatosis in NAFLD and contributing to inflammation, fibrosis, and progression to NASH and eventually hepatocellular carcinoma (HCC). Heat shock proteins (HSPs), including small HSPs such as HSP20 and HSP27, HSP60, HSP70, GRP78, and HSP90, are integral to cellular stress responses. They aid in protein folding, prevent aggregation, and facilitate degradation, thus mitigating cellular damage under stress conditions. In NAFLD, aberrant HSP expression and function contribute to disease pathogenesis. Understanding the specific roles of HSP subtypes in NAFLD offers insights into potential therapeutic interventions. This review discusses the involvement of HSPs in NAFLD pathophysiology and highlights their therapeutic potential. By elucidating the molecular mechanisms underlying HSP-mediated protection in NAFLD, this article aims to pave the way for the development of targeted therapies for this prevalent liver disorder.

## Introduction

Non-alcoholic fatty liver disease (NAFLD) encompasses a range of liver conditions where fat accumulates in the liver without excessive alcohol consumption. It spans from non-alcoholic fatty liver (NAFL), a milder form, to non-alcoholic steatohepatitis (NASH), which involves liver inflammation and damage, potentially progressing to fibrosis and cirrhosis. NAFLD often coexists with metabolic syndrome, obesity, type 2 diabetes, and dyslipidemia. While many cases are asymptomatic, NAFLD can lead to severe complications such as liver failure and cancer. Diagnosis involves imaging or biopsy, and treatment centers on lifestyle changes like diet and exercise, alongside medications and, in severe instances, weight loss surgery [1]. In recent studies, NAFLD has been renamed metabolic-associated steatotic liver disease (MASLD) to better reflect its underlying cause and remove stigmatizing language. The term “non-alcoholic” was seen as inadequate because it described the disease by exclusion rather than focusing on its primary driver, metabolic dysfunction. Additionally, the use of “fatty” was considered potentially stigmatizing. MASLD emphasizes the metabolic factors, such as obesity, diabetes, and insulin resistance, which contribute to the disease. The name change aligns with updated understanding and aims to improve awareness, reduce stigma, and facilitate research and treatment development [2–4]. NAFLD emerges from a complex network of molecular factors involving lipid handling, inflammation, oxidative stress, and mitochondrial dysfunction. The condition stems from an imbalance in how the liver manages lipids, with excess accumulation resulting from factors like heightened fat intake, internal lipid production, and fat release from adipose tissues, particularly worsened by insulin resistance. This lipid overload induces toxic effects, sparking inflammation and oxidative stress, which further damage liver cells. Concurrently, mitochondrial dysfunction disrupts energy production and promotes the generation of harmful reactive oxygen molecules. Disrupted processes like lipid droplet management and autophagy compound the problem, aggravating fat buildup. Interactions between the endoplasmic reticulum and mitochondria also influence lipid processing and cellular stress reactions. This intricate molecular landscape underpins the progression of NAFLD through its various stages, from simple fat accumulation to more severe conditions like NASH and liver cancer. Grasping these molecular intricacies holds the key to devising targeted treatments for NAFLD [5]. Endoplasmic reticulum (ER) stress plays a pivotal role in the pathogenesis of NAFLD, exerting multifaceted effects on various cell types within the liver. The unfolded protein response (UPR), initiated by ER stress, is activated in response to the accumulation of misfolded proteins within the ER. This activation triggers a cascade of events involving three main transmembrane sensors: inositol-requiring enzyme 1α (IRE1α), PKR-like ER kinase (PERK), and activating transcription factor 6 (ATF6). Dysregulation of these pathways, either independently or in conjunction, contributes to hepatic steatosis, inflammation, fibrosis, and ultimately, the progression to NASH and hepatocellular carcinoma (HCC). IRE1α, through its downstream target X-box binding protein 1 (XBP1), modulates lipid homeostasis, inflammation, and apoptosis, while PERK activation leads to translational repression and downstream activation of proapoptotic factors like C/EBP homologous protein (CHOP). Additionally, ATF6 regulates ER chaperone gene expression and influences lipid metabolism. ER stress also impacts hepatic stellate cells and Kupffer cells, contributing to fibrogenesis and inflammation in NAFLD. Understanding the intricate mechanisms of ER stress signaling in NAFLD holds promise for the development of targeted therapeutic interventions aimed at mitigating disease progression [6]. Heat shock proteins (HSPs) are a family of proteins that are produced by cells in response to exposure to stressful conditions, such as heat, toxins, or infections. They play a crucial role in maintaining cellular homeostasis by aiding in the proper folding of proteins, preventing protein aggregation, and facilitating the degradation of damaged proteins. Recently, there has been significant focus on HSPs due to their involvement in various diseases, including cancer, neurodegenerative disorders, and autoimmune diseases. Researchers are exploring their potential as therapeutic targets for treating these conditions, as well as their role in regulating immune responses and modulating cellular stress pathways. Moreover, HSPs are implicated in ER stress, a condition where unfolded or misfolded proteins accumulate in the ER, triggering a stress response. By assisting in protein folding and degradation, HSPs help alleviate ER stress and maintain ER function, highlighting their importance in cellular stress responses and disease pathology [7]. HSPs are critical regulators of cellular stress responses, including ER stress. HSPs such as HSP60 [8], HSP70 [9], GRP78 (a member of the HSP70 family) [10], and HSP90 [11] are chaperone proteins that assist in protein folding, prevent aggregation, and facilitate the degradation of misfolded proteins. In NAFLD, dysregulated expression of HSPs exacerbates disease progression by modulating key aspects of lipid metabolism, mitochondrial function, and inflammation [12].

Research has highlighted the potential of targeting HSPs as a therapeutic strategy in NAFLD. HSP modulators can restore proteostasis, alleviate ER stress, and reduce inflammatory responses [13]. Several compounds and strategies are currently being explored for their therapeutic potential in modulating HSPs in NAFLD. For example, phytochemicals like lipoic acid [14], abietic acid [15], and procyanidin B2 [16] have shown efficacy in reducing ER stress by modulating GRP78. These strategies offer promising avenues for therapeutic intervention by targeting the HSP pathways involved in NAFLD progression [9]. This study is to present an overview of the current understanding of the molecular causes, clinical implications, and therapeutic potential of biomarkers and targets in NAFLD.

## Heat shock protein classification

The classification HSPs encompasses a diverse array of proteins vital for cellular homeostasis under stress. Ranging from small HSPs (sHSPs) with molecular weights of 10 to 30 kDa to larger HSPs exceeding 100 kDa, these proteins are distributed across various cellular compartments. Small Heat Shock Proteins (HspBs) play crucial roles in embryonic development and cytoskeleton maintenance. HSP40 and HSP60 families participate in protein folding and assembly, with HSP60 implicated in numerous diseases. HSP70 and HSP90, located in the cytoplasm and nucleus, function as chaperones facilitating protein folding, transport, and degradation, with implications in disease pathogenesis. Finally, HSP100 and HSP110, found in the cytoplasm and nucleus, contribute to protein refolding and immune responses. This classification underscores the diverse roles of HSPs in cellular physiology and their potential as therapeutic targets in disease management. The numbers in the names of HSPs refer to the approximate molecular weight of the proteins in kilodaltons (kDa). For example, HSP40 proteins have a molecular weight around 40 kDa, and HSP60 proteins are around 60 kDa. These designations help to differentiate the proteins based on their size and function within the heat shock protein families [17]. Herein, we briefly review the HSPs that are involved in NAFLD (Table 1).

### Heat shock protein 20

HSP20, also referred to as heat shock protein beta-6 (HSPB6), belongs to the family of sHSP and holds a pivotal role in cellular equilibrium and stress management. Differing from other HSPs, HSP20 showcases a smaller size and operates independently of ATP for its chaperone activity. Structurally, it comprises a conserved alpha-crystallin domain (ACD) flanked by less conserved N-terminal and variable C-terminal regions. While HSP20 is typically expressed at basal levels across various tissues, its expression escalates in reaction to cellular stressors. Its primary function involves averting protein aggregation, sustaining protein stability, and aiding in the correct folding of proteins, thereby supporting proteostasis. Additionally, HSP20 has emerged as a pivotal regulator in a multitude of cellular mechanisms, including cell proliferation, apoptosis, and angiogenesis, rendering it a prospective therapeutic target in conditions such as cancer. Despite receiving comparatively less attention than other HSPs, growing evidence underscores its significance in cancer biology, underscoring the need for further exploration into its precise functions and therapeutic implications [18].

### Heat shock protein 27

HSP27, a member of the sHSP family, is a highly versatile molecular chaperone found across prokaryotes and eukaryotes. Structurally, it contains a conserved α-crystallin domain flanked by variable N and C-terminals, distinguishing it from other heat shock proteins. With molecular weights ranging from 16 to 42 kDa, HSP27 plays essential roles in cellular homeostasis and stress response by preventing protein aggregation and facilitating protein refolding. Beyond its chaperone function, HSP27 is involved in regulating cytoskeleton dynamics, cell cycle progression, and apoptosis. Its expression is induced by various stressors, and elevated levels have been observed in diseases such as cancer, where it contributes to increased invasiveness and drug resistance. Furthermore, HSP27 has implications in cellular networking, development, and aging, highlighting its importance as a potential biomarker and therapeutic target across diverse pathological conditions [19].

### Heat shock protein 60

HSP60 is a highly conserved molecular chaperone protein that plays a crucial role in maintaining cellular homeostasis, particularly under stress conditions encountered in cancer cells. Located predominantly in the mitochondria but also present in other cellular compartments, HSP60 functions in protein quality control by assisting in the correct folding of newly synthesized proteins, repairing misfolded proteins, and maintaining the stability of mitochondrial proteins. Its involvement in cancer is multifaceted, impacting various cellular processes including apoptosis, metabolism, proliferation, and metastasis. HSP60 interacts with a multitude of proteins to regulate tumor progression, demonstrating both anti-apoptotic and pro-apoptotic effects depending on its binding partners. Additionally, HSP60 influences metabolic reprogramming and proliferation signaling pathways and promotes tumor metastasis by modulating cellular migration and invasion. Its diverse functions suggest HSP60 as a potential biomarker and therapeutic target in human disease diagnosis and treatment [20].

### Heat shock protein 70

Hsp70, a highly conserved molecular chaperone, is a key player in maintaining protein homeostasis (proteostasis) within cells. Its multifunctional roles span from the folding of newly synthesized proteins to their degradation, ensuring their proper processing and preventing aggregation. Hsp70 operates through an intricate allosteric mechanism, where its activity is tightly regulated by nucleotide binding and substrate interaction. The chaperone cycle involves ATP-driven binding of Hsp70 to unfolded or misfolded proteins, followed by their release upon ATP hydrolysis. Co-chaperones, such as J-domain proteins (Hsp40s) and nucleotide exchange factors (NEFs), enhance Hsp70’s efficiency by stimulating ATP hydrolysis and facilitating substrate release. Hsp70’s substrate specificity, though broad, is influenced by cellular compartmentalization and the kinetics of substrate binding. Its ability to coordinate protein folding, prevent aggregation, and aid in disaggregation highlights its central role in maintaining cellular proteostasis. Dysregulation of Hsp70 function is implicated in various human pathologies, underscoring its importance in cellular physiology and disease mechanisms [21]. Glucose-regulated protein 78 (GRP78), also known as immunoglobulin heavy chain binding protein (BiP), is a crucial member of the HSP70 family, residing primarily on the ER membrane in eukaryotic cells. With its 654 amino acids, GRP78 plays a pivotal role in protein quality control by facilitating correct folding, assembly, and transport of proteins within the ER. Its expression increases under conditions of ER stress, such as glucose deprivation or disruptions in protein glycosylation or calcium storage. Structurally, GRP78 comprises two main domains: the ATP binding domain (ABD) at the amino-terminal and the substrate binding domain (SBD) at the carboxyl-terminal. While sharing homology with HSP70, GRP78 differs in its regulation of protein expression and sensitivity to certain stress inducers like cycloheximide. Functionally, GRP78 acts as a molecular chaperone, binding to misfolded proteins and initiating ER-associated degradation (ERAD) or unfolded protein response (UPR) pathways to restore cellular homeostasis. Additionally, GRP78 plays roles in embryo development, anti-apoptosis, and maintaining calcium balance in the ER. Under stress conditions, GRP78 can also translocate to the cell surface, where it functions as a receptor, influencing various cellular processes such as signaling, proliferation, migration, and apoptosis. Moreover, GRP78’s involvement in diseases like cancer and fungal infections, particularly in mediating the binding of pathogens like Rhizopus oryzae during cell invasion, underscores its significance in cellular physiology and pathology [22].

### Heat shock protein 72

Hsp72, a member of the heat shock protein family, plays a crucial role in cellular adaptation to stress, particularly heat stress. It is highly inducible in response to increased temperatures and functions as a molecular chaperone, assisting in the folding, assembly, and transport of proteins. Hsp72 also helps cells cope with various stressors by preventing protein misfolding and aggregation, inhibiting cellular apoptosis, and modulating inflammatory responses. Additionally, Hsp72 contributes to the development of thermotolerance, a state of increased resilience to heat stress following sublethal exposure to heat. Its accumulation within cells during heat acclimation is associated with improved whole-body tolerance to heat stress, suggesting that Hsp72 plays a critical role in promoting cellular and systemic adaptations that enhance survival in hot environments [23].

### Heat shock protein 90

HSP90, a member of the heat shock protein family, is a highly conserved molecular chaperone with a molecular weight of approximately 90-kDa. It plays a crucial role in cellular proteostasis by assisting in the folding of de novo synthesized or misfolded proteins, thereby preventing their aggregation and promoting their stability. Classified into various isoforms based on cellular localization, such as cytoplasmic (HSPC1, HSPC2, HSPC3), endoplasmic reticulum-resident (HSPC4 or GRP94), and mitochondrial (HSPC5 or TRAP1), HSP90 participates in fundamental cellular processes like apoptosis, cell cycle control, and signal transduction pathways. Furthermore, it is involved in regulating adaptive immunity and has been implicated in numerous pathological conditions, including cancer, viral infections, inflammation, and neurodegenerative diseases. HSP90’s structure comprises conserved domains—N-terminal, middle, and C-terminal—each fulfilling specific functions. The N-terminal domain binds ATP, the middle domain modulates ATPase activity and interacts with co-chaperones, and the C-terminal domain facilitates dimerization and interacts with client proteins. Through transcriptional regulation, posttranslational modifications, and interaction with co-chaperones, HSP90’s function is finely tuned to maintain cellular homeostasis and respond to stress conditions effectively [24].

**Table 1:** Classification of HSPs in NAFLD, a summary and comparison of their molecular, biochemical, and functional characteristics

HSP	Molecular Weight	Biochemical Properties	Molecular Mechanism	Functional Properties in NAFLD	Additional Notes	Ref.
**HSP20**	20 kDa	Small HSP, ATP-independent chaperone activity, prevents aggregation	Contains an α-crystallin domain; interacts with ERK2, inhibits phosphorylation	Regulates autophagy, suppresses autophagic activity, exacerbates lipotoxicity, promotes lipid accumulation and hepatocyte death under metabolic stress	Acts as a negative regulator of autophagy in hepatocytes, leading to increased lipid accumulation. Its expression increases with metabolic stress, particularly in high-fat diets.	[4, 8]
**HSP27**	16–42 kDa	Small HSP, phosphorylation regulates function, chaperone	Phosphorylated at serine residues (S15, S78, S82), promotes autophagy, affects STAT3/PKR pathway	Enhances autophagy, facilitates lipid clearance, reduces lipid accumulation, interacts with androgen signaling and improves insulin signaling	Phosphorylation alters its chaperone activity and helps promote autophagy, crucial for lipid metabolism under stress. Its interaction with androgen signaling pathways can influence NAFLD severity.	[25, 26]
**HSP60**	60 kDa	ATP-dependent chaperone, facilitates protein folding	Binds newly synthesized or misfolded proteins in mitochondria, promotes protein refolding	Regulates mitochondrial biogenesis and function, decreases weight gain, reduces adipose tissue, improves insulin sensitivity, suppresses mitochondrial dsRNA release and inflammation	Plays a protective role in NAFLD by improving mitochondrial function, reducing inflammation, and supporting fatty acid oxidation.	[27–29]
**HSP70**	70 kDa	ATPase activity; assists in protein folding, stabilization	Binds to unfolded proteins, works with co-chaperones (Hsp40s, NEFs), mediates proteostasis	Intracellular HSP70 reduces inflammation, extracellular HSP70 promotes inflammation; drives lipid metabolism, lipid accumulation, and ER stress in hepatocytes	Both intracellular and extracellular forms have opposite roles in inflammation; key in ER stress response and affects both lipid metabolism and the UPR pathway.	[25, 25, 25]
**HSP72**	72 kDa	Induced under stress, chaperone assists folding and assembly	Overexpression protects from oxidative stress and lipid accumulation, assists folding of misfolded proteins	Promotes fatty acid oxidation, decreases triglyceride storage, improves glucose tolerance, enhances mitochondrial function	Provides protective benefits in metabolic stress scenarios such as NAFLD by reducing oxidative damage, improving mitochondrial function, and regulating insulin resistance.	[12, 35]
**GRP78**	78 kDa	ER-resident chaperone, aids in protein folding and degradation	Two domains: ATP-binding domain (ABD) and substrate-binding domain (SBD); key regulator of unfolded protein response (UPR)	Key player in ER stress response, induces hepatic steatosis, involved in inflammation, apoptosis, and lipid accumulation during NAFLD progression	Highly involved in ER stress regulation, a key factor in NAFLD progression, through its ability to regulate UPR and assist in protein quality control.	[36–43]
**HSP90**	90 kDa	ATP-dependent molecular chaperone, stabilizes proteins	Interacts with client proteins, modulates protein stability and activity, regulates folding	Stabilizes key metabolic regulators (e.g., Akt, PPARγ), enhances lipogenesis, interacts with NLRP3 inflammasome, involved in insulin resistance and lipid metabolism	Plays multiple roles, from stabilizing lipid metabolism regulators (e.g., SREBPs) to interacting with inflammatory pathways, contributing to both lipid homeostasis and inflammation.	[11, 44–48]

## Small heat shock proteins in non-alcoholic fatty liver disease

### Heat shock protein 20

**Fig. 1:**
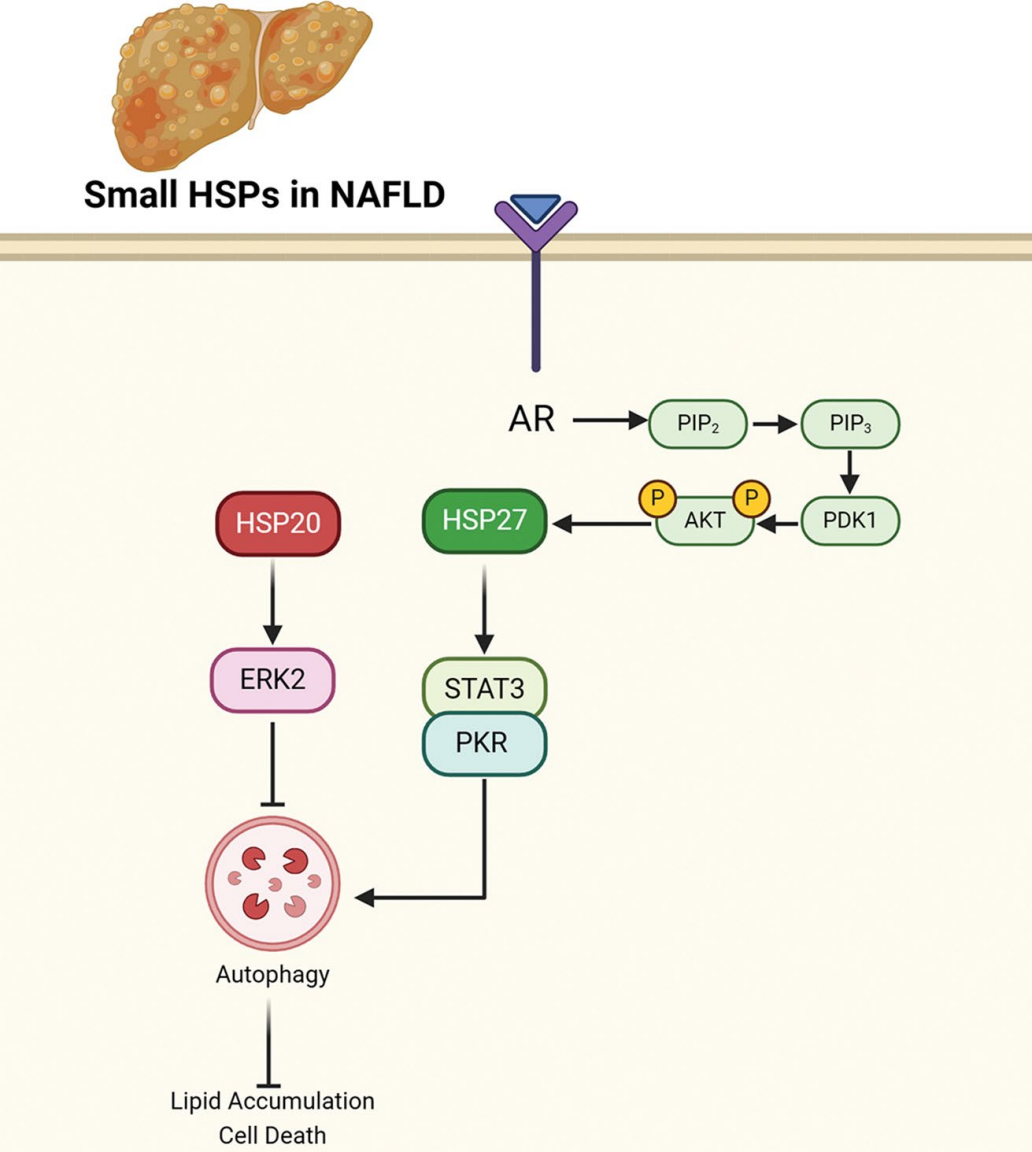
Role of small HSPs (HSP20 and HSP27) in NAFLD. The PI3K/Akt pathway positively induces HSP27 and activates autophagy, thereby preventing NAFLD progression. In contrast, HSP20 counteracts with autophagy and leads to cell death and lipid accumulation. Created with BioRender.com

HSP20 plays a critical role in regulating metabolic dysfunction-associated steatotic liver disease (MASLD) by modulating autophagy, particularly under conditions of metabolic stress such as high saturated fatty acid (SFA) exposure. In hepatocytes, the overexpression of HSP20 has been shown to exacerbate lipotoxicity by inhibiting autophagic processes. This inhibition of autophagy leads to increased cell death and lipid accumulation within hepatocytes, thereby contributing to the progression of MASLD. Interestingly, HSP20 also interacts with ERK2 and increases its phosphorylation, leading to suppression of autophagy. Inhibition of ERK1/2 phosphorylation mitigates the detrimental effects of HSP20 on lipotoxicity and autophagy. Conversely, knocking down HSP20 expression enhances autophagic activity, which helps reduce lipid accumulation and cell death, thus mitigating MASLD symptoms. Furthermore, in vivo studies indicate that mice with liver-specific overexpression of HSP20 fed a high-fat diet exhibit worsened metabolic parameters, including enhanced hepatic steatosis, increased liver damage, and impaired insulin signaling. These adverse effects are associated with disrupted autophagic signaling, evidenced by decreased levels of LC3II and increased levels of P62, markers of impaired autophagy. Therefore, HSP20 directly influences the severity and progression of MASLD by negatively regulating autophagic mechanisms in hepatocytes, highlighting its potential as a therapeutic target for managing MASLD (Fig. 1) [4].

### Heat shock protein 27

Hsp27 plays a multifaceted role in the pathogenesis of NAFLD. In the context of NAFLD, Hsp27 emerges as a critical player in regulating hepatic lipid metabolism and autophagy. Research indicates that Hsp27 undergoes phosphorylation, particularly at serine residues 15, 78, and 82, in response to various stress stimuli. Phosphorylated Hsp27 exhibits altered cellular functions, including anti-apoptotic effects, modulation of cell cycle progression, and regulation of cytoskeletal dynamics. Importantly, phosphorylated Hsp27 is implicated in promoting autophagy, a cellular process crucial for maintaining lipid and energy homeostasis during stress conditions. In NAFLD models, such as high-fat diet-fed mice for 4 to 16 weeks and palmitate-treated hepatic cells, Hsp27 phosphorylation is induced, leading to enhanced autophagy and improved hepatic lipid clearance. Mechanistically, phosphorylated Hsp27 disrupts the STAT3/PKR complex, resulting in increased eIF2α phosphorylation and subsequent activation of autophagy. This interaction between Hsp27 and STAT3 is pivotal for promoting autophagy-mediated lipid clearance in hepatocytes. Furthermore, inhibition of Hsp27 phosphorylation exacerbates hepatic steatosis in NAFLD models, underscoring the protective role of phosphorylated Hsp27 against lipid accumulation in the liver. Overall, Hsp27 emerges as a promising therapeutic target for NAFLD, and further elucidation of its regulatory mechanisms may offer novel strategies for the management of this prevalent metabolic disorder [26]. In NAFLD, androgen signaling through the androgen receptor (AR) plays a crucial role in regulating the expression of Hsp27. Activation of AR by androgens such as dihydrotestosterone (DHT) leads to the induction of Hsp27 expression in androgen-sensitive liver cells. This induction of Hsp27 is mediated by the PI3K/Akt pathway, as evidenced by DHT-induced Akt phosphorylation, particularly in AR-positive liver cell lines. Additionally, suppression of Hsp27 expression inhibits DHT-induced cell cycle arrest, suggesting a functional role for Hsp27 in mediating the effects of androgen/AR signaling on cell cycle regulation in NAFLD. Furthermore, the involvement of the PKR/eIF2α pathway, independent of ER stress response, in mediating DHT-induced Hsp27 expression underscores the complex interplay of signaling pathways in androgen-mediated regulation of cellular processes in NAFLD (Fig. 1) [25].

## Heat shock protein 60 in non-alcoholic fatty liver disease

**Fig. 2:**
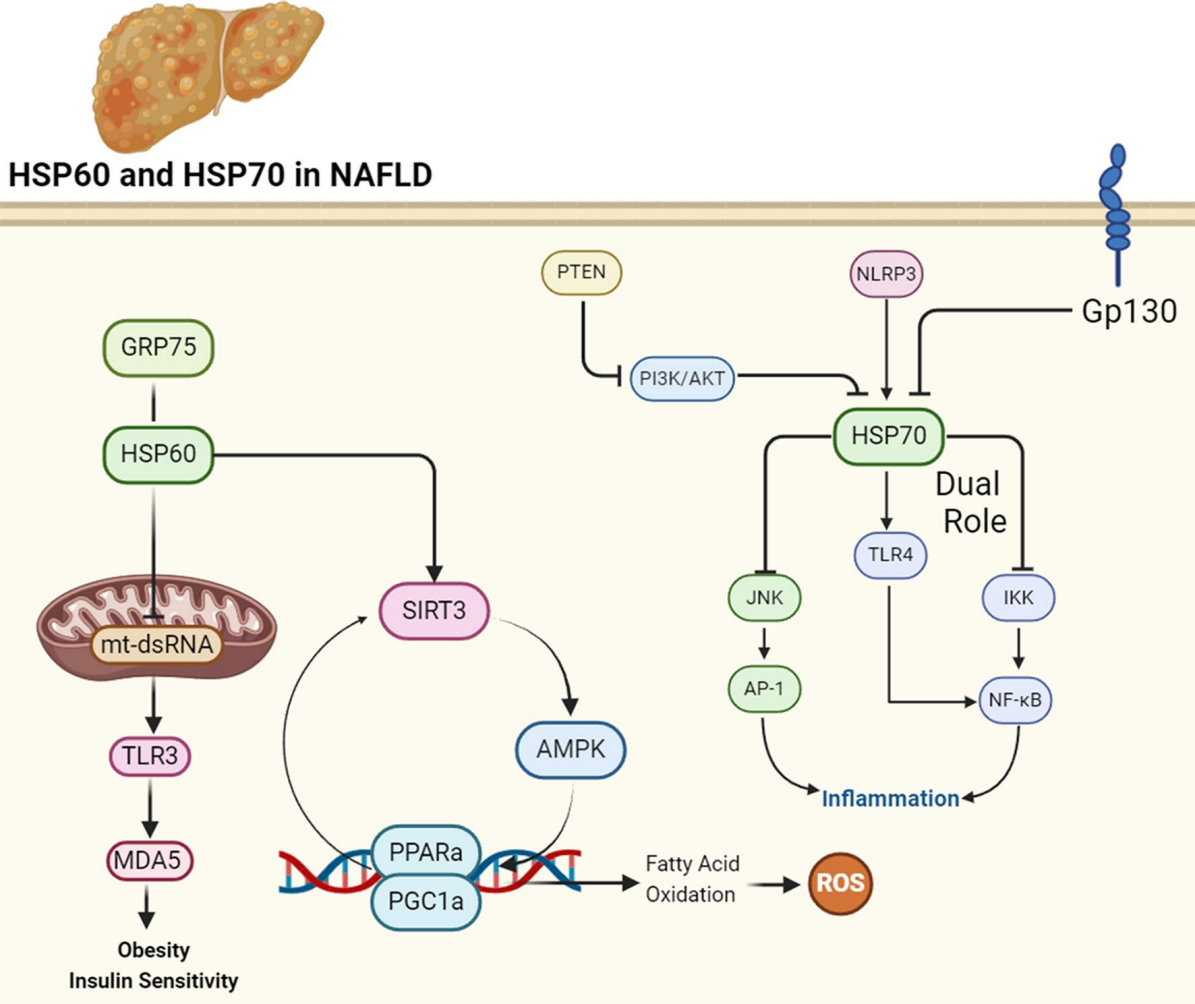
Regulatory function of HSP60 and HSP70 in NAFLD. HSP60 is protective against NAFLD progression by inhibiting fat formation and increasing insulin sensitivity. On the other hand, the function of HSP70 is double-edged, both supporting and hindering NAFLD progression. Created with BioRender.com

HSP60 mitigates several key aspects of NAFLD pathogenesis. Firstly, it regulates lipid metabolism by reducing weight gain, adipose tissue accumulation, and hepatic steatosis induced by a high-fat diet (HFD). Additionally, HSP60 enhances glucose tolerance and insulin sensitivity, addressing metabolic dysregulation associated with NAFLD. Importantly, HSP60 overexpression suppresses the release of mitochondrial double-stranded RNA (mt-dsRNA), thereby mitigating inflammation mediated by the mt-dsRNA/TLR3/MDA5 pathway. Moreover, HSP60 modulates mitochondrial biogenesis, attenuating mitochondrial DNA content perturbations induced by HFD. These findings collectively highlight HSP60 as a potential therapeutic target for NAFLD, offering avenues for intervention by regulating lipid metabolism, inflammation, and mitochondrial function [28]. A significant decrease in HSP60 levels in human fatty liver specimens and in mice with diet-induced obesity (DIO) is observed, suggesting a potential link between HSP60 and metabolic disorders. Through various experiments, including transgenic overexpression and knockdown studies, the study elucidates several key functions of HSP60 in NAFLD. Firstly, HSP60 overexpression mitigates obesity, improves glucose tolerance, and enhances insulin sensitivity in mice fed with HFD. Additionally, HSP60 represses HFD-induced adiposity, inflammation, and oxidative stress in both liver and adipose tissues. Mechanistically, HSP60 promotes fatty acid oxidation and inhibits lipid accumulation in hepatocytes, partly through SIRT3-mediated signaling pathways involving AMPK/PGC1α/PPARα. These findings suggest that HSP60 plays a crucial role in regulating lipid metabolism and may serve as a potential therapeutic target for NAFLD by modulating mitochondrial function and metabolic pathways [8]. Grp75, also known as glucose-regulated protein 75, is a protein involved in the formation of mitochondria-associated endoplasmic reticulum membranes (MAMs), which play a crucial role in regulating lipid metabolism and cellular adaptation to environmental changes. In the context of NAFLD, Grp75 expression is found to be decreased in hepatocytes, particularly under conditions of high-fat high-sucrose diet-induced obesity. This reduction in Grp75 expression is associated with alterations in MAM integrity and mitochondrial dynamics, leading to disruptions in lipid homeostasis. Interestingly, decreased Grp75 expression is linked to an increase in heat shock protein family D (HSPD1), also known as Hsp60, which is a marker of mitochondrial stress (Fig. 2). This suggests that Grp75 may regulate HSP60 expression, potentially through its role in maintaining MAM integrity, and thereby modulate mitochondrial function and lipid metabolism in NAFLD [29].

## Heat shock protein 70 in non-alcoholic fatty liver disease

HSP70, induced in response to cellular stress, exhibits a complex role wherein intracellular HSP70 demonstrates anti-inflammatory properties, while extracellular HSP70 is implicated in pro-inflammatory processes associated with insulin resistance. In NAFLD, HSP70 expression is upregulated, particularly in obese individuals, suggesting its involvement in metabolic disturbances. Experimental evidence, both in vivo and in vitro, underscores the significant impact of HSP70 on hepatic lipid metabolism. HSP70 overexpression promotes lipid accumulation in hepatocytes, accompanied by the upregulation of lipogenic genes, while HSP70 knockdown leads to reduced lipid accumulation and decreased expression of lipogenic enzymes. These findings illuminate the intricate role of HSP70 in driving hepatic steatosis through the stimulation of lipogenesis, highlighting its potential as a therapeutic target for managing NAFLD and related metabolic disorders [9]. Low birth weight, induced by intrauterine growth restriction (IUGR), exacerbates molecular changes associated with hepatocarcinogenesis when combined with fructose consumption in adulthood. Rats with low birth weight and exposed to fructose exhibit increased hepatic myeloperoxidase (MPO) activity, AKT phosphorylation, and serum aspartate transaminase (AST) levels. Moreover, markers of cell proliferation, including Cyclin D, PCNA, HGF, and Hspa4/Hsp70 expression, as well as Ki-67 positive cell counts, are elevated in their livers. Additionally, insulin-like growth factor 1 (IGF-1) levels are reduced in these rats. These findings collectively suggest that the combination of low birth weight and fructose consumption intensifies the molecular alterations associated with NASH and HCC, indicating a complex interplay between prenatal factors and postnatal dietary habits in shaping liver health and disease susceptibility [30, 31]. Zhao et al. examined the relationship between HSPA8 single-nucleotide polymorphism (SNP), serum heat shock cognate 71 kDa protein (HSC70) concentration, and carotid artery atherosclerosis in NAFLD patients. Results indicated that patients with the major allele genotype of HSPA8 exhibited significantly higher serum HSC70 concentrations compared to those with at least one copy of the minor allele. Furthermore, stratification by sex revealed a significant association between HSPA8 genotype and carotid intima–media thickness (IMT) specifically in male patients, where individuals with at least one copy of the minor allele displayed significantly greater IMT compared to those homozygous for the major allele. These findings suggest a potential link between HSPA8 SNP, serum HSC70 levels, and the development of carotid artery atherosclerosis in male NAFLD patients, highlighting the intricate genetic and physiological factors contributing to cardiovascular risk in this population [49]. Particularly, Tang et al. focused on single nucleotide polymorphisms (SNPs) in calcium transporter genes (ITPR2, VDAC1) and related chaperones (HSPA5, HSPA9, SIGMAR1, CANX, PPID) in the Chinese population. Their findings revealed significant associations between certain SNPs and NAFLD susceptibility, highlighting the potential role of genetic variations in Ca^2+^ transport proteins in disease pathogenesis. Notably, specific SNPs were found to be associated with either increased or decreased risk of NAFLD, suggesting a complex interplay between genetic predisposition and disease development. Moreover, stratified analyses elucidated gender and age-specific effects, while a combined factor model integrating genetic and metabolic factors demonstrated good predictive accuracy for NAFLD risk [32].

### Regulation of nuclear factor κB and c-Jun NH2-terminal kinases signaling pathways

The progression of NAFLD to NASH is characterized by increased inflammation and cellular stress, where key signaling pathways such as nuclear factor κB (NF-κB) and c-Jun NH2-terminal kinases (JNK) play central roles. In this context, the HSP70 pathway is crucial in mitigating these inflammatory processes. HSP70, known for its chaperone function, exerts anti-inflammatory effects by inhibiting NF-κB activation and JNKs, thereby suppressing inflammatory signaling cascades (Fig. 2). However, obesity disrupts this protective mechanism by downregulating HSP70 expression. In obese individuals, chronic inflammation and elevated levels of circulating fatty acids contribute to the dysregulation of metabolic tissues, including the liver. As a result, decreased HSP70 expression leads to increased susceptibility to inflammatory insults and metabolic dysfunction, facilitating the progression from NAFLD to NASH. Understanding the interplay between obesity, inflammation, and HSP70 regulation is critical for developing targeted therapies to manage NASH and its associated complications [13].

### Regulation by nucleotide-binding domain-like receptor protein 3

Nucleotide-binding domain-like receptor protein 3 (NLRP3), a critical component of the inflammasome complex, plays a significant role in regulating the immune response and inflammatory events in various diseases, including MASLD. In MASLD, NLRP3 activation triggers the inflammatory cascade, contributing to hepatocyte damage and inflammation. One of the key pathways influenced by NLRP3 in MASLD is HSP70 -toll-like receptor 4 (TLR4) axis. HSP70, known for its role in immune response modulation, interacts with NLRP3, influencing its activation status. Upon activation, NLRP3 physically interacts with HSP70, exacerbating the inflammatory response in the liver. Additionally, HSP70 acts as a ligand for TLR4, further activating the nuclear factor kappa-light-chain-enhancer of activated B cells (NF-κB) pathway, leading to enhanced transcription of genes associated with inflammation (Fig. 2) [3].

### Regulation by phosphatase and tensin homolog

The role of phosphatase and tensin homolog (PTEN) in oxidative stress and DNA damage in NAFLD has been investigated. Elevated insulin levels, common in obesity and early type 2 diabetes mellitus (T2DM), lead to mitochondrial dysfunction and increased production of reactive oxygen species (ROS), potentially contributing to DNA damage and cancer risk. The PI3 kinase/AKT pathway, regulated by PTEN, plays a crucial role in this process. In vitro experiments using hepatocyte cell lines demonstrate that insulin treatment induces ROS production and DNA damage, which is exacerbated by PTEN inhibition. Similarly, Pten haplodeficient mice fed a high-fat diet exhibit increased hepatic triglyceride content and upregulated expression of fatty acid synthase, confirming the role of PTEN in metabolic dysregulation. Furthermore, the mice show elevated expression of HSP70 and HO-1, markers of oxidative stress, and increased phosphorylation of AKT, indicating enhanced insulin signaling. Genomic damage, assessed by γ-H2AX staining, is heightened in the liver tissues of mice fed a high-fat diet, suggesting a potential link between PTEN deficiency, insulin signaling, and genomic instability in the development of liver pathologies such as cancer. These findings highlight the critical role of PTEN in the pathogenesis of NAFLD, shedding light on the intricate interplay between metabolic dysregulation, oxidative stress, and genomic damage in liver diseases associated with obesity and insulin resistance (Fig. 2) [34].

### Regulation by glycoprotein 130

Gp130, a transmembrane protein, serves as a receptor component for interleukin-6 (IL-6) family cytokines. In the context of NAFLD, gp130 has been implicated in modulating cellular responses to oxidative stress and mitochondrial dynamics. Specifically, in a cellular model of steatohepatitis induced by tBHP/Oleic treatment, the addition of gp130 influences the expression of HSP70, a crucial cytoprotective molecule. A study revealed that gp130 supplementation decreases HSP70 gene expression in HepG2 cells subjected to oxidative stress, suggesting a potential regulatory role of gp130 in mitigating cellular stress responses in NAFLD. This finding underscores the intricate interplay between gp130 signaling and cellular stress pathways, highlighting its relevance in the pathogenesis of NAFLD and potential implications for therapeutic interventions targeting HSP70-mediated cytoprotection in liver disease (Fig. 2) [33].

## 78 kDa glucose-regulated protein in non-alcoholic fatty liver disease

As mentioned earlier, GRP78 is a subgroup of HSP70 family. The function of GRP78 has been studied very well in NAFLD. GRP78, a marker for endoplasmic reticulum stress (ERS), emerges as a pivotal player in the pathogenesis of NAFLD. GRP78 is upregulated in various stages of NAFLD, particularly in NASH, indicating its involvement in disease progression. Its expression correlates positively with key markers of NAFLD severity, including inflammation, hepatocyte apoptosis, and macrophage infiltration. In animal models and cell culture studies, GRP78 upregulation is associated with ER stress and plays a crucial role in the pathological processes underlying NAFLD, suggesting it as a potential therapeutic target for mitigating disease progression and associated complications [36]. Long-term exposure to a high-sucrose diet has been found to down-regulate hepatic ER stress adaptive pathways while concurrently potentiating de novo lipogenesis (DNL) through the modulation of GRP78. This phenomenon was observed in a study involving mice exposed to high-sucrose diets from weaning to young adulthood. Initially, the high-sucrose diet-induced adaptive unfolded protein response pathways mediated by GRP78, which aimed to restore ER homeostasis. However, prolonged exposure to the high-sucrose diet led to a transition of ER stress towards a proapoptotic pattern, characterized by the down-regulation of UPR sensors and chaperones, including GRP78. This dysregulation of ER stress adaptive pathways coincided with increased expression of genes involved in DNL, such as carbohydrate response element-binding protein (ChREBP) and stearoyl-CoA desaturase 1 (SCD1), ultimately promoting hepatic lipid accumulation. These findings highlight the intricate interplay between dietary factors, ER stress, and lipid metabolism, with GRP78 serving as a key mediator in the pathogenesis of NAFLD under conditions of long-term high-sucrose intake [37]. Long-term consumption of a high-fat diet (HFD) during early ages induces significant metabolic alterations and hepatic damage, as evidenced by increased adiposity, impaired glucose management, and elevated levels of plasma leptin and non-esterified fatty acids (NEFA). These effects are accompanied by hepatic lipid accumulation and morphological changes indicative of steatosis and fibrosis. Moreover, HFD promotes angiogenesis and alters the expression of factors involved in inflammation, such as vascular endothelial growth factor receptor 2 (VEGF-R2), angiopoietin-like 4 (ANGPTL-4), and cluster of differentiation 36 (CD36). The up-regulation of CD36, a protein with pro-inflammatory properties, is associated with increased expression of GRP78-BiP and phosphorylated eukaryotic translation initiation factor 2 alpha (p-EIF2α), suggesting activation of endoplasmic reticulum stress and pro-inflammatory pathways. Additionally, elevated levels of interleukin-6 (IL-6) mRNA further support the presence of hepatic inflammation in response to HFD. These findings underscore the detrimental effects of early-life HFD consumption on metabolic health and liver function, highlighting the importance of dietary interventions in preventing obesity-related complications such as NAFLD [38]. In a mouse model of alcohol and high-fat diet-induced liver injury, excess iron exacerbates ER stress-associated pathways, contributing to the progression of liver damage. The study reveals that increased hepatic iron levels lead to dysregulation of ER homeostasis, resulting in enhanced ER stress markers such as CCAAT/enhancer binding protein-homologous protein (CHOP) and phosphorylated protein kinase double-stranded RNA-dependent-like ER kinase (p-PERK). Despite the activation of ER stress pathways, downstream effects such as phosphorylation of eukaryotic initiation factor 2-α (p-EIF2α) are not observed, indicating a potential disconnect between the UPR initiation and attenuation phases. Additionally, iron overload augments the expression of autophagy-related genes, suggesting a compensatory mechanism to alleviate ER stress. However, impaired autophagic flux is observed, characterized by accumulation of p62 and decreased levels of microtubule protein 1 light chain 3 (LC3-I and LC3-II), indicating compromised autophagic activity. These findings highlight how excess iron disrupts ER homeostasis and autophagy, contributing to liver injury in the context of alcohol and high-fat diet-induced liver disease [39, 40].

### GRP78 regulators in non-alcoholic fatty liver disease

#### Hypoxia-inducible factor 1-alpha

**Fig. 3:**
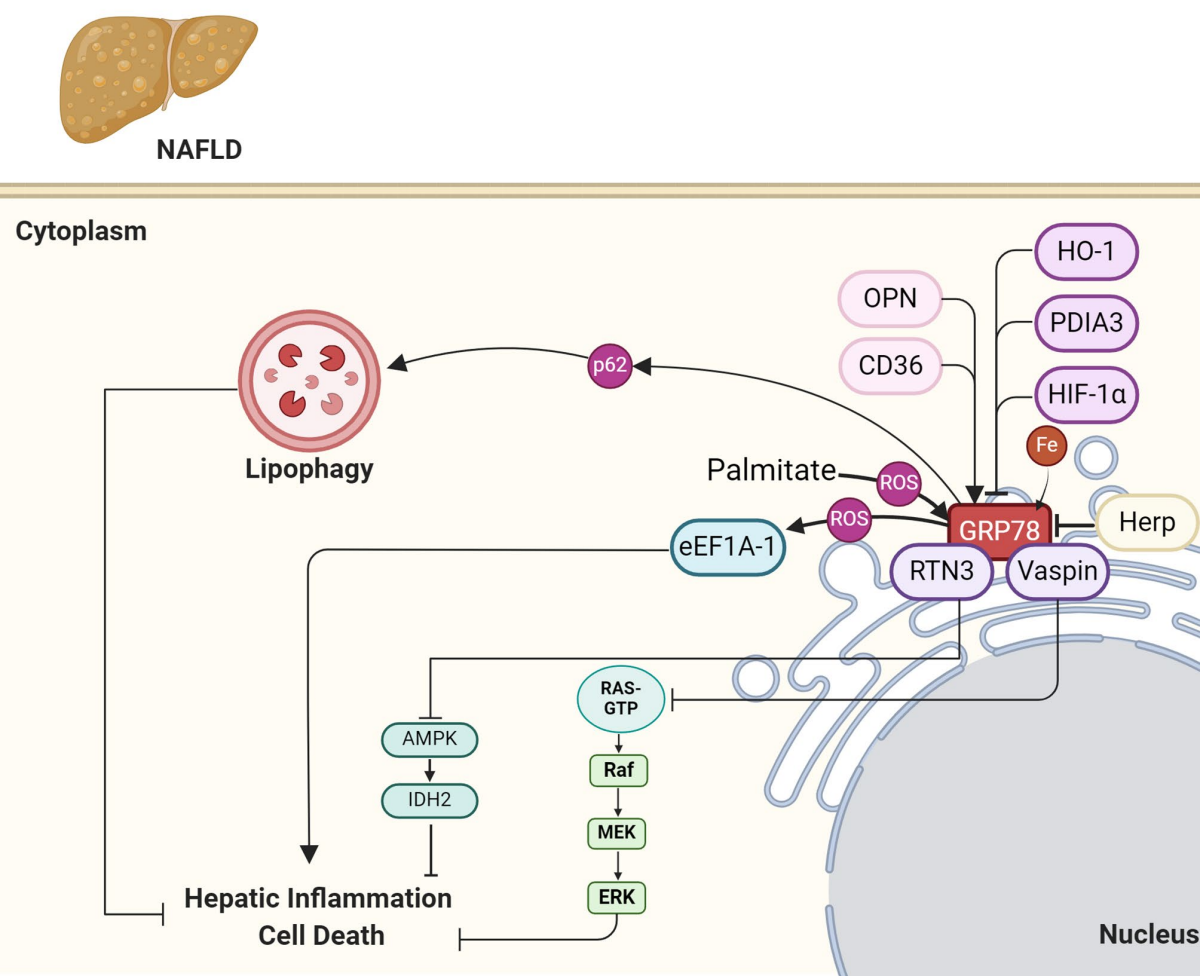
Molecular interactions of GRP78 in NAFLD. Activation of GRP78 can induce hepatic cell inflammation and death via multiple mechanisms. Molecules such as HO-1, PDIA3, HIF-1a, and Herp can inhibit its activity and prevent NAFLD progression, while OPN and CD63 exacerbate the disease at the cellular level. On the other hand, GRP78 can interact with proteins, including RTN3 and Vaspin, which can potentiate NAFLD progression via MAPK and AMPK pathways. Created with BioRender.com

Hypoxia-inducible factor 1-alpha (HIF-1α) regulates the expression of the ER stress marker GRP78 through a direct mechanism. It has been observed that overexpression of HIF-1α attenuated the palmitic acid-induced induction of GRP78, while knockdown of HIF-1α resulted in increased GRP78 expression. This suggests that HIF-1α plays a role in modulating ER stress by regulating GRP78 expression levels. Additionally, the study further elucidated the mechanism underlying this regulation by demonstrating that HIF-1α directly interacts with the CHOP promoter, inhibiting CHOP expression, which may indirectly affect GRP78 expression since CHOP is known to induce GRP78 during ER stress (Fig. 3) [41].

#### Eukaryotic elongation factor 1 A-1

The eukaryotic elongation factor 1 A-1 (eEF1A-1) is involved in various cellular processes, including protein synthesis and actin cytoskeleton dynamics, and has been implicated in oxidative and ER stress responses. The role of eEF1A-1 in NAFLD, particularly focusing on its response to lipotoxic stress induced by palmitate, a saturated fatty acid implicated in NAFLD progression has been explored. In HepG2 cells, exposure to palmitate led to a modest increase in eEF1A-1 protein levels and concurrent ER stress, as evidenced by increased GRP78 expression. Interestingly, eEF1A-1 partially relocalized from the ER to newly polymerized actin at the cell periphery, preceding cell death. Chemical inhibition of eEF1A-1 peptide elongation function with didemnin B did not prevent palmitate-induced ER stress initiation but decreased subsequent cell death, suggesting a role for eEF1A-1 in promoting lipotoxicity. Additionally, in obese ob/ob mice with severe hepatic steatosis and ER stress, liver eEF1A-1 protein levels were increased. These findings suggest that eEF1A-1 may contribute to the progression of NAFLD by promoting hepatocyte lipotoxicity, potentially through its functions in protein synthesis and actin cytoskeleton dynamics (Fig. 3) [42].

#### Protein disulfide-isomerase A3

In NAFLD, PDIA3, an ER-resident chaperone, appears to play a regulatory role in modulating the expression of GRP78, a key marker of ERS. When hepatocytes are exposed to elevated levels of saturated fatty acids, such as palmitic acid, PDIA3 expression increases, likely as part of the UPR aimed at restoring ER homeostasis. Silencing PDIA3 exacerbates hepatocellular steatosis and apoptosis, suggesting its protective role in NAFLD. Mechanistically, PDIA3 knockdown leads to a significant upregulation of GRP78, along with other ERS markers such as PERK and CHOP. This suggests that PDIA3 may act as a negative regulator of GRP78 expression, possibly by fine-tuning the UPR signaling pathway. The precise molecular mechanisms underlying this regulatory relationship between PDIA3 and GRP78 warrant further investigation but hold promise for understanding and potentially targeting ER stress in NAFLD (Fig. 3) [43].

#### C-X-C motif chemokine receptor 3

The role of C-X-C Motif Chemokine Receptor 3 (CXCR3) in the pathogenesis of steatohepatitis, particularly in the transition from simple steatosis to more severe forms, has been investigated. CXCR3 expression was found to be significantly upregulated in human NAFLD liver tissues as well as in murine models of steatohepatitis induced by dietary and genetic factors. Mechanistically, CXCR3 appears to exacerbate hepatic inflammation and injury by promoting macrophage infiltration, T cell immune responses, and the expression of pro-inflammatory cytokines and chemokines. Moreover, CXCR3 activation induces hepatic lipogenesis while suppressing autophagy, leading to lipid accumulation and impaired lipid metabolism. Furthermore, CXCR3 contributes to the activation of the ubiquitin-proteasome system (UPS) and ER stress, thereby aggravating hepatic injury and apoptosis. Importantly, inhibition of CXCR3, either by genetic deletion or pharmacological antagonists, attenuates hepatic steatosis, inflammation, and injury in murine models of steatohepatitis. These findings suggest that targeting CXCR3 signaling pathways may hold therapeutic potential for the treatment of steatohepatitis and its associated complications [50].

#### Heme oxygenase-1

Heme oxygenase-1 (HO-1) plays a crucial role in regulating the expression of GRP78. Through experimental models and in vitro studies, it has been observed that HO-1 induction, achieved through treatment with hemin, leads to a reduction in hepatic steatosis and inflammation while concurrently suppressing ER stress. This effect is evidenced by decreased expression of GRP78, among other ER stress markers, in the liver tissue of NASH models following HO-1 induction. Conversely, inhibition of HO-1 exacerbates liver injury and ER stress, accompanied by elevated expression of GRP78. Additionally, in hepatocyte cell lines, overexpression of HO-1 reduces reactive oxygen species (ROS) levels and attenuates ER stress, thereby downregulating GRP78 expression. These findings suggest that HO-1 exerts its hepatoprotective effects in NASH, at least in part, by modulating ER stress and GRP78 expression, highlighting HO-1 as a potential therapeutic target for NASH management (Fig. 3) [51].

#### Osteopontin

A study demonstrated that osteopontin (OPN) plays a significant role in regulating GRP78 in NAFLD. OPN deficiency led to decreased levels of GRP78 in the liver, particularly in 10-month-old mice, indicating a potential link between OPN and GRP78 expression. GRP78 is an important chaperone protein involved in ER homeostasis, and its decrease is associated with ER stress, a key factor in NAFLD progression. The study also found that OPN deficiency induced senescence-associated hepatosteatosis, suggesting that OPN may regulate GRP78 expression through mechanisms involving cellular senescence and ER stress pathways. These findings underscore the role of OPN in maintaining liver homeostasis and suggest that its regulation of GRP78 may contribute to the pathogenesis of NAFLD (Fig. 3) [52].

#### Homocysteine-induced ER protein

Homocysteine-induced ER protein (Herp) is a stress-response protein located in ER membrane, known for its involvement in ERS. In a study utilizing a high-fat diet-induced NAFLD mouse model, Herp knockout was found to offer protection against NAFLD progression. Herp knockout resulted in decreased body weight gain, liver weight increase, and serum levels of triglycerides, total cholesterol, and inflammatory markers such as alanine aminotransferase (ALT) and aspartate aminotransferase (AST). Furthermore, Herp knockout ameliorated insulin resistance, hepatic steatosis, inflammation, fibrosis, and hepatocyte apoptosis. These protective effects were attributed to the reduction of ERS, as evidenced by decreased expression of ERS markers such as Grp78, Chop, and Atf4. Notably, Herp knockout mediated the downregulation of Grp78, suggesting a potential mechanism through which Herp exerts its protective role in NAFLD (Fig. 3) [53].

#### Vaspin

Vaspin, an adipokine released by various tissues including adipocytes and liver cells, has been implicated in metabolic diseases such as NAFLD. While the exact role of vaspin in NAFLD remains elusive, recent studies shed light on its potential mechanisms. Vaspin acts as a ligand for the cell surface receptor GRP78 in endothelial and ovarian cells, activating protein kinase A (PKA) and mitogen-activated protein kinase (MAPK) signaling pathways. GRP78, a multifunctional chaperone protein primarily located in the endoplasmic reticulum, plays a crucial role in protein folding, assembly, and transport, as well as in the regulation of cellular stress responses. In NAFLD, vaspin’s interaction with GRP78 and activation of PKA and MAPK pathways are thought to modulate cellular metabolism and inflammation, leading to improvements in lipid accumulation, metabolic function, and inflammatory response. This suggests that targeting the vaspin-GRP78 axis, along with MAPK signaling, could hold promise as a therapeutic approach for NAFLD by regulating key pathways involved in the disease’s pathogenesis (Fig. 3) [54].

#### Reticulon 3

Reticulon 3 (RTN3) is a protein primarily located in the ER, playing various roles in cellular functions. Recent research has uncovered its involvement in NAFLD, a prevalent hepatic disorder characterized by fat accumulation in the liver. Analysis across multiple datasets has shown elevated levels of RTN3 in NAFLD patients, high-fat diet mice, and cell lines exposed to oxidized low-density lipoprotein (ox-LDL), suggesting a potential correlation between RTN3 expression and NAFLD progression. Transgenic mouse models overexpressing RTN3 exhibit significant fat accumulation in the liver, accompanied by elevated liver enzymes and triglyceride levels, indicating a direct role of RTN3 in promoting NAFLD development. Mechanistic studies have revealed that RTN3 interacts with GRP78. This interaction inhibits the AMPK-isocitrate dehydrogenase 2 (IDH2) pathway, leading to mitochondrial dysfunction, increased ROS production, and ultimately NAFLD progression. This suggests that RTN3 regulates GRP78 function, disrupting cellular metabolic pathways and promoting hepatic lipid accumulation, highlighting its significance as a potential therapeutic target for NAFLD treatment (Fig. 3) [55].

#### 10 N-degron pathway

The N-degron pathway regulates Bip/GRP78 through a series of molecular interactions and modifications. Specifically, this pathway involves the enzymatic conjugation of the amino acid L-Arg to the N-termini of Bip/GRP78 by ATE1-encoded R-transferases, resulting in the generation of the N-degron Nt-Arg. This Nt-Arg serves as a recognition signal for p62, an N-recognin protein that binds Nt-Arg and other N-degrons. Upon binding, p62 undergoes a conformational change, exposing its PB1 domain and facilitating cargo condensation through self-polymerization. This complex formation between p62 and Nt-arginylated Bip/GRP78 leads to the recruitment of autophagic membranes to sites of lipophagy, ultimately initiating the degradation of lipid droplets (LDs). Through these molecular interactions, the N-degron pathway plays a central role in regulating the degradation of Bip/GRP78 and its involvement in lipophagy-mediated lipid degradation processes [56].

## Heat shock protein 72 in non-alcoholic fatty liver disease

HSP72 has emerged as a crucial factor in mitigating NAFLD-associated metabolic disturbances. Studies indicate that induction of HSP72 through interventions like heat treatment or pharmacological means can effectively improve glucose tolerance, reduce hepatic insulin resistance, and decrease triglyceride storage in the liver. Additionally, HSP72 induction has been linked to enhancements in mitochondrial integrity and function, which are vital for maintaining hepatic lipid metabolism. Direct modulation of HSP72 expression in primary hepatocytes further demonstrates its role in preserving mitochondrial integrity and promoting fatty acid oxidation, thus potentially preventing lipid accumulation in the liver. Consequently, targeting HSP72 presents a promising strategy for mitigating hepatic insulin resistance, NAFLD progression, and ultimately type 2 diabetes [12]. Hsp72, induced under stress conditions, plays a crucial role in protecting the liver against injury in NAFLD by mitigating hepatocellular death, oxidative stress, and JNK signaling. It has been indicated that Hsp72 overexpression in transgenic mice attenuates liver injury induced by toxins like DDC and APAP, as well as lipotoxicity from a methionine-choline-deficient (MCD) diet, common models for NAFLD. In these models, Hsp72 reduces hepatocellular death, as evidenced by lower levels of alanine transaminase (ALT), reduced oxidative stress, and decreased activation of c-Jun N-terminal kinase (JNK), a key regulator of cell death pathways. The protective effects of Hsp72 involve mechanisms such as decreased phosphorylation of JNK and its downstream targets, including c-Jun and receptor-interacting serine/threonine-protein kinase 3 (Rip3) [57]. In addition, the researchers explored the role of Growth arrest and DNA damage-inducible 45β (GADD45β) in NAFLD. They found that GADD45β expression was significantly reduced in both NAFLD patients and a murine model of NAFLD induced by a high-fat, high-fructose (HFHFr) diet. Overexpression of GADD45β mitigated HFHFr-induced lipid accumulation, decreased serum triglyceride levels, and improved insulin resistance in mice. Mechanistically, GADD45β was found to interact directly with HSP72, a stress-inducible protein, to prevent its degradation via the proteasome pathway. Further experiments revealed that HSP72 knockdown reversed the beneficial effects of GADD45β on insulin sensitivity and lipid metabolism. Additionally, the study demonstrated a positive correlation between GADD45β and HSP72 expression in clinical databases and in the liver tissue of HFHFr-induced NAFLD mice. These findings suggest that GADD45β may serve as a potential therapeutic target for NAFLD through its interaction with HSP72 to regulate insulin sensitivity and lipid metabolism [35].

## Heat shock protein 90 in non-alcoholic fatty liver disease

With new diagnostic criteria in place, MASLD encompasses a broader spectrum of patients at risk of metabolic comorbidities compared to the previous terminology, NAFLD. Despite its prevalence, diagnosing and assessing the severity of MASLD remains challenging due to limited biomarkers, with current options like ALT and cytokeratin 18 (CK-18) fragment showing unsatisfactory predictive value. Heat shock protein 90 alpha (Hsp90α), involved in inflammation regulation, emerges as a potential biomarker candidate, with studies indicating its elevation in NAFLD, although its role in MASLD remains unclear. Additionally, the therapeutic potential of teprenone (geranylgeranylacetone, GGA), known for its anti-inflammatory effects, is explored in this context. In a clinical study involving MASLD patients and healthy controls, elevated serum Hsp90α levels correlated positively with metabolic parameters. A predictive model combining Hsp90α, BMI, HbA1c, and ALT demonstrated high accuracy in identifying MASLD. In cellular and animal models, GGA was found to upregulate Hsp90α expression and ameliorate steatosis, inflammation, and insulin resistance in MAFLD. Moreover, increased nuclear transporting of Hsp90α was observed in MASLD mice, suggesting a potential mechanism underlying its involvement in the disease process. These findings shed light on the diagnostic potential of Hsp90α and the therapeutic efficacy of GGA in MASLD, offering new insights and avenues for further research in this complex metabolic disorder [44]. In addition, serum levels of Hsp90β and total Hsp90 were significantly higher in overweight and obese children compared to healthy controls, while Hsp90α levels showed no significant difference. Hsp90β levels tended to be higher in insulin-resistant patients, suggesting a potential association with insulin resistance. Furthermore, significant differences in Hsp90α and Hsp90β levels were observed between NAFLD and non-NAFLD patients, with higher Hsp90β and lower Hsp90α levels in NAFLD patients. The ratio between Hsp90α and Hsp90β showed promising diagnostic accuracy for NAFLD, indicating its potential as a reliable biomarker for this condition in overweight and obese children. These findings suggest that Hsp90 isoforms may play a role in the pathogenesis of NAFLD and could serve as valuable diagnostic indicators in this population [11].

### Molecular function of Hsp90β

#### Regulation of Akt-GSK3β-FBW7 Axis

**Fig. 4:**
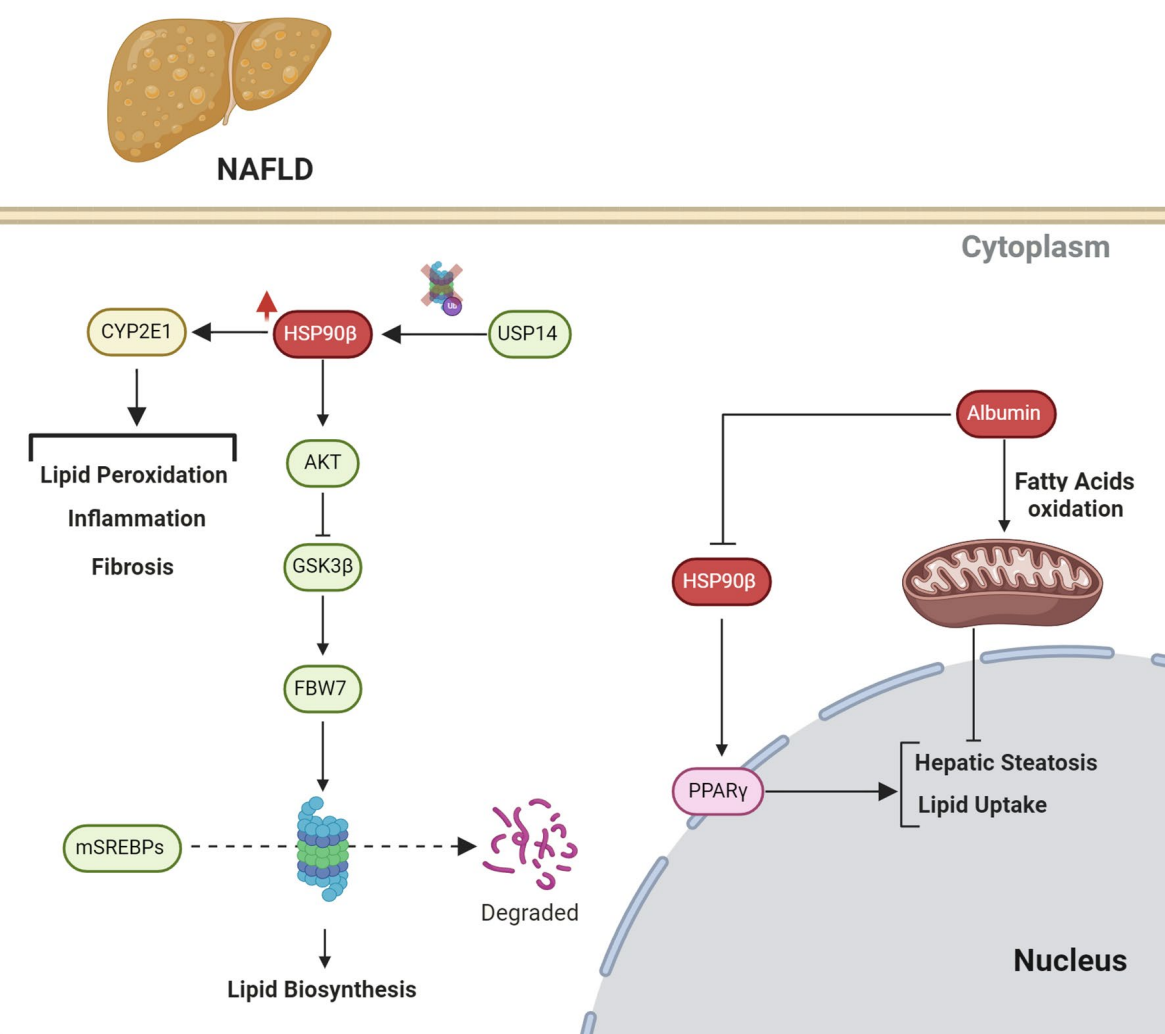
HSP90 molecular interactions in NAFLD. HSP90β orchestrates dysregulated lipid homeostasis by stabilizing mature SREBPs through modulation of the Akt-GSK3β-FBW7 pathway, leading to enhanced lipogenesis. Additionally, HSP90 interacts with PPARγ, promoting its stability and transcriptional activity, thereby exacerbating hepatic steatosis. Furthermore, HSP90AA1 stabilization by USP14 enhances CYP2E1-mediated lipid peroxidation and inflammation. Albumin interacts with HSP90 to maintain mitochondrial homeostasis and protect against oxidative stress in NAFLD. The mitochondria play a crucial role in preventing hepatic steatosis by promoting fatty acid oxidation (FAO), where fatty acids are broken down for energy, reducing lipid accumulation in the liver. Albumin enhances this process by acting as a carrier for fatty acids and facilitating their transport to the mitochondria for oxidation, helping maintain mitochondrial homeostasis and cellular energy balance. Additionally, albumin interacts with HSP90, stabilizing its function and protecting against oxidative stress, further mitigating mitochondrial dysfunction and fatty acid accumulation in NAFLD. Targeting these interactions presents potential therapeutic avenues for managing NAFLD by ameliorating aberrant lipid accumulation and oxidative stress in the liver. Created with BioRender.com

HSP90β emerges as a pivotal regulator of lipid metabolism through its modulation of the Akt-GSK3β-FBW7 pathway. Elevated HSP90β levels in NAFLD contribute to dysregulated lipid homeostasis by promoting the stabilization of mature SREBPs (mSREBPs), key transcription factors involved in de novo lipid synthesis. Mechanistically, HSP90β interacts with Akt, a central kinase in the insulin signaling pathway, thereby facilitating Akt phosphorylation and activation. Subsequently, activated Akt phosphorylates GSK3β, inhibiting its activity and preventing the phosphorylation-dependent degradation of mSREBPs by the ubiquitin ligase FBW7. This intricate interplay orchestrated by HSP90β ultimately leads to the accumulation of mSREBPs and enhanced transcription of lipogenic genes, resulting in increased de novo synthesis of fatty acids and cholesterol. The upregulation of these lipogenic pathways contributes to lipid accumulation within hepatocytes, exacerbating lipid disorders such as NAFLD. Targeting HSP90β with selective inhibitors such as corylin disrupts this pathway, promoting the ubiquitination and proteasomal degradation of mSREBPs and consequently ameliorating lipid dysregulation in NAFLD (Fig. 4) [45].

#### Regulation of peroxisome proliferator-activated receptor Gamma

PPARγ (peroxisome proliferator-activated receptor gamma) is a nuclear receptor that plays a central role in regulating lipid metabolism and adipogenesis. In the context of NAFLD, PPARγ is implicated in promoting lipid storage and exacerbating hepatic steatosis. Hsp90 acts as a molecular chaperone that facilitates the folding, stability, and function of various client proteins, including PPARγ. In NAFLD, heightened ER stress triggers an upregulation of Hsp90, which, in turn, stabilizes and enhances the activity of PPARγ. This interaction between Hsp90 and PPARγ promotes the transcriptional activity of PPARγ, leading to increased expression of its target genes involved in lipid uptake and storage. Inhibition of Hsp90 activity disrupts this interaction, resulting in reduced PPARγ signaling and amelioration of hepatic steatosis. Thus, targeting Hsp90-mediated modulation of PPARγ presents a potential therapeutic approach for managing NAFLD by mitigating aberrant lipid accumulation in the liver (Fig. 4) [46].

#### Regulation by Ubiquitin Specific Peptidase 14

USP14, a proteasome-associated deubiquitinating enzyme, plays a crucial role in regulating protein degradation through the ubiquitin-proteasome system (UPS). In the context of NAFLD, USP14 emerges as a key player in disease progression. It interacts with HSP90AA1, a molecular chaperone involved in protein folding and stability, to modulate the stability of cytochrome p4502E1 (CYP2E1), a critical enzyme in NAFLD pathogenesis. Through deubiquitination of lysine 48 (K48) linkages on HSP90AA1, USP14 prevents its degradation by the proteasome, thereby enhancing HSP90AA1-mediated stabilization of CYP2E1. This stabilization promotes hepatic lipid peroxidation (LPO), inflammation, and fibrosis, contributing to the progression of NAFLD to NASH. Both in vitro and in vivo studies demonstrate that modulation of USP14 expression influences NAFLD severity, highlighting its potential as a therapeutic target. This intricate regulatory mechanism underscores the importance of understanding the role of UPS components in hepatic lipid metabolism and oxidative stress in liver disease progression (Fig. 4) [47].

#### Regulation by Albumin

Albumin plays a crucial role in maintaining mitochondrial homeostasis and inhibiting NAFLD through its interaction with HSP90. Essentially, albumin acts as a carrier for various molecules including fatty acids, bilirubin, and drugs, thereby aiding in their transport and distribution throughout the body. In the context of mitochondrial homeostasis, albumin ensures proper mitochondrial function by facilitating the transport of fatty acids to the mitochondria for oxidation, which helps in maintaining cellular energy balance. Moreover, albumin has been shown to directly interact with HSP90, a molecular chaperone protein involved in protein folding and stabilization. This interaction enhances the stability and function of HSP90, leading to improved mitochondrial function and protection against oxidative stress. In NAFLD, decreased levels of albumin have been associated with mitochondrial dysfunction and impaired fatty acid oxidation, contributing to the development and progression of the disease. By promoting the stability and activity of HSP90, albumin helps mitigate these effects, thereby exerting a protective effect against NAFLD (Fig. 4) [48].

## Therapeutic strategies for targeting heat shock proteins in non-alcoholic fatty liver disease

Therapeutic targeting of HSPs holds significant importance in managing NAFLD due to their pivotal roles in cellular protection, protein homeostasis, and stress response mechanisms. HSPs, including HSP70 and HSP90, help fold and refold proteins, thereby ensuring cellular stability under stress conditions such as oxidative stress and inflammation, which are prevalent in NAFLD. Additionally, HSPs modulate inflammatory pathways and apoptosis, processes integral to the progression of NAFLD to more severe stages like NASH and fibrosis. Given their central role in mitigating stress-induced damage and regulating cellular metabolism, HSPs represent a promising target for therapeutic intervention. Modulating HSP activity can potentially restore proper protein function, alleviate mitochondrial dysfunction, and reduce inflammatory responses, thereby preventing or slowing liver disease progression in NAFLD patients (Table 2).

### Phytochemicals

Phytochemicals offer promising therapeutic avenues for NAFLD due to their diverse biological activities. These natural compounds, such as flavonoids and other plant-derived metabolites, can target various aspects of NAFLD pathogenesis. They regulate lipid metabolism by activating key enzymes and pathways like AMPK, which reduces lipid accumulation in the liver [58, 59]. Additionally, many natural substances alleviate oxidative stress and inflammation, two critical factors in the progression of NAFLD to more severe forms like NASH. They do this by enhancing the body’s antioxidant defenses through mechanisms that include activating the Nrf2 pathway, which helps in reducing oxidative damage and inflammatory responses in hepatic cells. Thus, the multifaceted effects of natural products on lipid metabolism, oxidative stress, and inflammation make them potential candidates for the prevention and treatment of NAFLD [60].

#### Lipoic acid

(+)-Lipoic acid, also known as alpha-lipoic acid (ALA), is a naturally occurring compound that functions as a powerful antioxidant and is involved in various metabolic processes, particularly in mitochondrial energy production. In NAFLD, ALA has been shown to have protective effects, partly through its interaction with HSPs. HSPs, including HSP60 and HSP90, play crucial roles in protein folding and protecting cells against stress-induced damage. In studies involving liver cells subjected to fatty acid-induced stress (a model for NAFLD), ALA treatment resulted in the modulation of these HSPs, helping to reduce protein misfolding and mitigate cellular stress. By influencing the expression and function of HSPs, ALA helps restore mitochondrial function and improves the cellular response to unfolded proteins, which are critical aspects in preventing and managing NAFLD. This mechanism underscores the therapeutic potential of ALA in enhancing mitochondrial resilience and protecting liver cells from the lipid and oxidative stress commonly seen in NAFLD [14].

#### Hugan Qingzhi

- Hugan Qingzhi tablet (HQT) is a traditional Chinese medicine formula comprised of Rhizoma alismatis, Fructus crataegi, Pollen typhae, Folium nelumbinis, and Radix notoginseng, with a history of use in alleviating NAFLD. Recent research has elucidated its protective mechanisms, particularly its ability to modulate endoplasmic reticulum (ER) stress, a key contributor to NAFLD progression. HQT effectively attenuates ER stress through downregulation of GRP78, a marker protein associated with ER stress response. In vivo studies in rat models demonstrate that HQT treatment leads to reduced body weight, liver index, serum lipid levels, and inflammatory cytokines, along with improved liver histopathology and ER structure. Moreover, in vitro experiments reveal that HQT-mediated serum can effectively alleviate ER stress in liver cells. These findings underscore HQT’s potential as a therapeutic agent for NAFLD, highlighting its ability to mitigate ER stress and hinder disease progression through GRP78 modulation both in vivo and in vitro [61].

#### Isoquercitrin

Isoquercitrin (IQ) is a flavonoid compound known for its anti-inflammatory properties and is found abundantly in various plants. In NAFLD, IQ has shown promising potential in hindering disease progression. Through experimental models, including the MCD diet-induced mice model, IQ demonstrated significant effects in reducing hepatic lipid accumulation and inflammation. Notably, it exerts its anti-inflammatory effects by targeting the NLRP3 inflammasome, a key mediator of liver inflammation in NAFLD. IQ suppresses the activation of the NLRP3 inflammasome by downregulating the expression of HSP90, a crucial protein involved in the inflammasome activation process. This inhibition ultimately leads to a reduction in liver inflammation and fibrosis, highlighting IQ’s potential as a therapeutic agent for NAFLD by modulating inflammatory pathways and oxidative stress [62].

#### Abietic acid

Abietic acid (AA) is a compound found in water-processed rosin, historically used in Asian medicine for treating conditions like psoriasis. Recent research indicates its potential therapeutic role in mitigating the progression of NAFLD. AA demonstrates anti-inflammatory properties and has been shown to reduce bacterial activity. In the context of NAFLD, AA exhibits several molecular mechanisms that contribute to its protective effects. Firstly, AA dose-dependently suppresses lipid accumulation in hepatocytes, likely by downregulating lipogenesis-related proteins. Secondly, AA attenuates ER stress, a key contributor to NAFLD development, by reducing the expression of ER stress markers and apoptosis in hepatocytes. This effect is mediated, at least in part, through the activation of AMPK, a cellular energy regulator known to alleviate ER stress. Additionally, AA selectively increases the expression of the ER chaperone ORP150, which further contributes to the suppression of lipid accumulation, ER stress, and apoptosis in hepatocytes. Importantly, the induction of ORP150 expression by AA is dependent on AMPK signaling. These findings suggest that AA holds promise as a therapeutic agent for NAFLD by targeting multiple pathways involved in its pathogenesis, including lipid metabolism and ER stress, through the activation of AMPK and the induction of ORP150 expression [15].

#### Procyanidin B2

Procyanidin B2 (PCB2), derived from herbal cinnamon, demonstrates promising therapeutic potential in alleviating palmitic acid (PA)-induced injury in HepG2 cells, particularly through modulation of the ERS pathway. PA, a saturated fatty acid, is implicated in hepatocyte injury associated with NAFLD. The study reveals that PCB2 protects HepG2 cells from PA-induced damage by improving cell viability, inhibiting apoptosis, and mitigating oxidative stress and calcium disequilibrium. PCB2’s protective effects are associated with its ability to alleviate ERS, as evidenced by reduced expression of ERS markers such as glucose-regulated proteins 78 and 94, phosphorylated PERK, eIF2α, IRE1α, and CHOP. Moreover, PCB2 suppresses ERS-mediated NLRP3 inflammasome activation, thereby attenuating inflammation in PA-exposed HepG2 cells. These findings suggest that PCB2 holds promise as a therapeutic agent for NAFLD by targeting the ERS pathway to mitigate PA-induced hepatocyte injury [16].

#### Plant Sterol Ester of α-Linolenic acid

Plant Sterol Ester of α-Linolenic Acid (PS-ALA) is a compound synthesized from plant sterol and α-linolenic acid, with known cholesterol-lowering properties. This study investigates its potential impact on the progression of NAFLD, a prevalent chronic liver condition. The research focuses on PS-ALA’s effect on NAFLD progression via the modulation of GRP78, a marker of ER stress. Results indicate that PS-ALA treatment significantly reduces liver and body weight, attenuates lipid accumulation, and suppresses the expression of GRP78, a key indicator of ER stress. By mitigating ER stress, PS-ALA demonstrates promise in ameliorating NAFLD progression, suggesting its potential as a therapeutic intervention for this condition [63].

#### Tanshinone IIA

Tanshinone IIA (Tan IIA) is a lipophilic diterpene derived from the traditional herbal medicine Salvia miltiorrhiza Bunge, known for its therapeutic properties in various conditions including cardiovascular disorders, Alzheimer’s disease, and liver fibrosis [64]. In NAFLD, Tan IIA shows promise in hindering disease progression. Through its antioxidant properties and ability to attenuate ER stress, Tan IIA effectively counteracts the detrimental effects of palmitate-induced lipotoxicity. By reducing ER stress markers such as GRP78, ATF6, phospho-eIF2α, and CHOP, Tan IIA mitigates hepatocyte apoptosis, steatosis, and cytotoxicity induced by palmitate in HepG2 cells. These findings suggest that Tan IIA may offer therapeutic benefits for NAFLD by targeting ER stress-induced apoptosis and lipid accumulation, potentially presenting a novel avenue for treatment intervention in this prevalent liver disorder [65].

#### Jiang-Zhi Granules

Jiang-Zhi Granules (JZG) is a traditional herbal remedy composed of Gynostemma pentaphyllum (Thunb.) Makino, Polygoni cuspidati rhizoma, Artemisia capillaris Thunb, Salviae Miltiorrhizae Radix, and Folium Nelumbinis. It has been utilized clinically to treat fatty liver and hyperlipidemia, showing promising results in improving liver function and reducing hepatosteatosis in NAFLD models induced by high-fat diet. Recent studies have delved into its potential mechanisms of action, revealing its ability to modulate the endoplasmic reticulum stress (ERS) signaling pathway. Specifically, JZG administration leads to a reduction in the expression of GRP78, a central regulator of ERS. GRP78 upregulation is associated with ERS induction, and its downregulation by JZG suggests a protective effect against ERS-mediated liver injury in NAFLD. Thus, JZG holds promise as a therapeutic intervention for NAFLD, potentially through its modulation of ERS pathways, particularly the attenuation of GRP78 expression [66].

#### Ginsenoside Rg1

Ginsenoside Rg1, derived from ginseng, is a natural compound that exhibits promising therapeutic potential against NAFLD. Through a series of experiments utilizing a HFD-induced NAFLD mouse model, Rg1 demonstrated several beneficial effects. Firstly, it effectively regulated body weight and liver weight, controlling obesity associated with NAFLD. Additionally, Rg1 significantly reduced serum transaminase levels and blood lipid levels, indicative of its ability to mitigate liver inflammation and inhibit hepatic fat synthesis. Importantly, Rg1 exhibited antioxidant properties, promoting lipid peroxidation and enhancing antioxidant capacity, thus protecting against oxidative stress, a common feature of NAFLD. Moreover, Rg1 promoted fatty acid oxidation while inhibiting triglyceride synthesis, partly through upregulating the expression of peroxisome proliferator-activated receptor-alpha (PPARα). Furthermore, Rg1 inhibited the expression of genes associated with ER stress, such as GRP78, as well as genes involved in inflammasome activation, contributing to its anti-inflammatory effects [67].

#### 10 Schisandra Chinensis extract

Schisandra Chinensis extract, derived from a traditional herbal medicine, exhibits potential in impeding the progression of NAFLD by targeting endoplasmic reticulum (ER) stress. NAFLD, characterized by excessive liver lipid accumulation, is closely linked to metabolic disorders. Schisandra Chinensis extract demonstrates pharmacological activities including anti-inflammatory, antioxidant, and hepatoprotective effects. Through in vitro and in vivo experiments, it is revealed that the extract effectively inhibits ER stress and reduces intracellular triglyceride levels, key contributors to NAFLD development. Molecular mechanisms elucidate that Schisandra Chinensis extract downregulates the expression of ER stress markers such as GRP78 and CHOP, consequently mitigating hepatic lipid accumulation and inflammation associated with ER stress. Moreover, the extract’s active component, schisandrin, emerges as a significant contributor to its protective effects against ER stress-induced hepatic steatosis. These findings suggest the potential of Schisandra Chinensis extract as a therapeutic agent for NAFLD, offering insights into its molecular mechanisms and potential for future drug development [68].

#### 11 melatonin

Melatonin, a hormone primarily produced by the pineal gland, exhibits diverse physiological functions, including regulation of circadian rhythms, antioxidant activity, and modulation of metabolic processes. In NAFLD, melatonin has emerged as a potential therapeutic agent due to its ability to mitigate oxidative stress, improve liver function, and regulate metabolic parameters. One of the key mechanisms through which melatonin exerts its effects in NAFLD is by modulating the expression of GRP78, melatonin supplementation was found to significantly reduce the expression of GRP78, indicating a potential role in alleviating ER stress. This suggests that melatonin may help restore ER homeostasis and mitigate the pathological consequences of ER stress in NAFLD, highlighting its therapeutic potential in the management of this condition [69].

#### 12 Docosahexaenoic

Docosahexaenoic acid (DHA) is a polyunsaturated fatty acid (PUFA) found abundantly in marine fish oil and is essential for mammals as it cannot be synthesized in the body. In NAFLD, emerging evidence suggests that DHA plays a crucial role in mitigating disease progression. Studies have shown that NAFLD, often associated with high fructose consumption, involves dysregulated lipid metabolism and ER stress. DHA supplementation has been found to alleviate NAFLD by multiple mechanisms. Firstly, DHA reduces lipid accumulation in hepatocytes exposed to fructose, thereby attenuating hepatic steatosis. It achieves this by suppressing de novo lipogenesis while promoting fatty acid oxidation. Furthermore, DHA exerts its protective effects by modulating ER stress pathways, thereby mitigating fructose-induced ER stress response. DHA treatment reduces the expression of ER stress markers such as GRP78, IRE1α, and XBP-1, consequently alleviating ER stress in hepatocytes. Additionally, DHA regulates the expression of hepatic lipid-homeostasis regulators, including transcription factors involved in de novo lipogenesis, leading to reduced lipid synthesis and enhanced lipid oxidation [70].

#### 13 coffee

Coffee consumption appears to confer protective effects against liver damage induced by a HFD through various mechanisms. Firstly, it reduces serum alanine aminotransferase (ALT) levels, indicative of liver injury, and lowers serum triglyceride levels, thus attenuating hepatic steatosis. Histological analysis reveals that coffee administration diminishes liver steatosis, inflammation, and fibrosis induced by the HFD. At a molecular level, coffee consumption modulates liver protein expression, upregulating certain proteins involved in stress response pathways and downregulating others. Specifically, it enhances the expression of ER chaperones such as GRP78, which are crucial for protein folding and ER stress management. Increased expression of mitochondrial chaperones is also observed, suggesting improved mitochondrial function and stress response. Furthermore, coffee consumption reduces lipid peroxidation and DNA oxidative damage, indicating a decrease in oxidative stress within the liver. Collectively, these findings suggest that coffee consumption exerts protective effects against liver damage induced by an HFD by alleviating hepatic steatosis, inflammation, and fibrosis, and modulating stress response pathways including upregulation of GRP78, thus highlighting its potential therapeutic role in mitigating NAFLD progression [71].

#### 14 Emodin

Emodin, a naturally occurring anthraquinone compound found in Rheum palmatum L., possesses potent anti-inflammatory, antioxidative, and hepatoprotective properties. Recent studies have highlighted its potential in mitigating NAFLD, a condition characterized by hepatic steatosis, particularly induced by high-fructose diets. Emodin demonstrates protective effects against NAFLD by modulating lipid metabolism, as evidenced by its ability to attenuate hepatic steatosis and lipid accumulation in fructose-fed rats. Mechanistically, emodin exerts its influence by downregulating the expression of key lipogenic genes (ACC, FAS, SCD1) while restoring levels of the fatty acid oxidation enzyme CPT1. Moreover, emodin interacts with the UPR pathway, specifically targeting GRP78, a chaperone protein pivotal in UPR activation. Emodin’s modulation of GRP78 expression ultimately alleviates ERS, highlighting its multifaceted approach in combating NAFLD pathogenesis [72].

#### 15 Hesperetin

Hesperetin is a flavonoid compound predominantly found in citrus fruits, renowned for its anti-inflammatory, antioxidant, and cytoprotective properties. In NAFLD, hesperetin has shown promising results by modulating the UPR, particularly through the regulation of the ER chaperone protein GRP78. The activation of GRP78 by hesperetin helps mitigate ERS, which is a critical factor in the pathogenesis of NAFLD. Studies indicate that hesperetin activates protective pathways within the UPR, such as sXBP1 and GRP78, which can counteract the lipotoxic effects caused by saturated fatty acids like palmitate. This regulation not only helps prevent cell death but also reduces the severity of liver damage in NAFLD, making hesperetin a potential therapeutic agent for managing and treating this condition. However, it’s important to note that the protective effects of hesperetin are contingent on its ability to induce GRP78, highlighting the significance of this protein in hesperetin’s mechanism of action against NAFLD [73]. In another study, it was shown that Hesperetin suppresses HFD-induced body weight gain and hepatic steatosis in rats, although it doesn’t affect the serum lipid profile significantly. HSP treatment decreases the expression of key ERS markers such as GRP94, ATF6, ATF4, and phosphorylated PERK and IRE1α in rat liver tissues and human THP-1 macrophages, leading to a reduction in inflammation-inducing cytokines like IL-1β, IL-6, and TNF-α. Furthermore, Hesperetin inhibits hepatic lipid synthesis proteins, suggesting that it may curtail lipid accumulation by hindering ERS. Thus, HSP acts through a multifaceted mechanism involving the reduction of ERS and inflammation, key factors contributing to the development and progression of NAFLD [74].

#### 16 silymarin

Silymarin, extracted from the seeds of the milk thistle plant (Silybum marianum), is a complex mixture primarily composed of flavonolignans, notably silybin. This natural product exhibits diverse biological activities, including anti-inflammatory, antidiabetic, anticancer, and antifibrotic effects. In NAFLD, silymarin shows promise as a therapeutic agent due to its ability to modulate crucial pathways implicated in disease progression. One such pathway is the ER stress response, where silymarin’s effects are notable. Silymarin has been found to alleviate ER stress by enhancing the proper folding capacity of proteins and reducing the accumulation of misfolded proteins within the ER. Specifically, silymarin downregulates the expression of ER stress markers such as GRP78 and XBP-1, thereby attenuating ER stress-induced liver damage. Additionally, silymarin regulates genes associated with lipid metabolism, leading to reduced hepatic inflammation, insulin resistance, and lipid accumulation. Through its multifaceted effects on ER stress and lipid metabolism pathways, silymarin emerges as a promising candidate for inhibiting NAFLD progression and offering potential therapeutic benefits for this increasingly prevalent liver disorder [10].

**Table 2:** Treatment strategies for targeting HSP proteins in NAFLD

Treatment	Drug type	HSP Family	Other target genes	Pathway	Model	Highlights	Ref.
Phytochemicals							
Lipoic acid	Organosulfur compound derived from caprylic acid (octanoic acid)	(HSP90 and HSP60)↑	CLPP GPX1, GSTP1, GSR, GSH, and HO-1	-	In Vitro (HepG2 cell line)	↓Mitochondrial unfolded protein response ↓Oxidative stress and aging ↑ Antioxidant response and HO-1	[14]
Hugan Qingzhi	Traditional Chinese medicine formula	GRP78↓	PERK, p-PERK, ATF6	PERK	In Vivo (Sprague-Dawley rats)	↓ Body weights ↑ Triglycerides, total cholesterol and very-low-density lipoprotein ↓ High-density lipoprotein cholesterol levels were significantly decreased.	[61]
Isoquercitrin	Quercetin glycosides	HSP90↓	SGT1	HSP90-NlLRP3 pathway	In Vitro (AML-12 hepatocytes) and In Vivo (MCD mouse model)	↓ Inflammation ↓ AST and ALT	[62]
Abietic acid	Water-processed rosin	GRP78↓	SREBP1 and SCD1	AMPK/ORP150 signaling	In Vitro (Human primary hepatocytes (palmitate-treated)	↓ Hepatic lipid buildup, apoptosis, and ER stress	[15]
Procyanidin B2	Extracted from herbal cinnamon	GRP78↓	p-IKKα/β, p-NF-κB p65, NLRP3	NLRP3/caspase 1/IL-1β	In Vitro (HepG2)	Protects hepatic cells from palmitic acid injury ↓ Apoptosis ↓ERS and calcium disequilibrium	[16]
Plant Sterol Ester of α-Linolenic Acid (PS-ALA)	α-Linolenic Acid ester	GRP78↓	Srebp-1c, Fasand, Srebp-2 and Hmgcr	Ire1α/Xbp1s signal pathway and NLRP3	In Vivo (C57BL/6J mice)	↓ Inflammatory cytokines and transaminase ↓ ER Stress-Induced Oxidative Stress and mitochondrial dysfunction	[63]
Tan IIA	lipophilic diterpene extracted from Danshen	GRP78↓	ATF6	PERK/eIF2α and TTF6 pathways	In Vitro (HepG2)	Blocks palmitate-induced cell death and steatosis ↓ER Stress	[65]
Jiang-Zhi Granule	Granule extracted from Jiang-Zhi	GRP78↓	PERK, EIF2α, and NFκB	GRP78/PERK/EIF2α/NF-κB	In Vivo (Wistar rats)	↓ Inflammation, necrosis and hepatocyte vacuolar degeneration ↑ Liver function Improve the serum lipid and hepatosteatosis	[66]
Ginsenoside Rg1	Monomer in ginseng	GRP78↓	NLRP3, IL-1β, and IL-18	NLRP3	In Vitro (mouse liver tissue) and In Vivo (C57BL/6 mice)	↓ Body weight and liver wet weight ↓Serum transaminase and blood lipid	[67]
Schisandra chinensis extract	Schisandra chinensis (Turcz.) Baill. (SC)	GRP78↓	CHOP, and XBP-1	Nrf2, JNK, and NFκ-B	In Vitro (HepG2 cells) and In Vivo (tunicamycin-injected mice)	↓ER stress and intracellular triglyceride level ↓ ER stress-associated inflammation	[68]
Melatonin	Indoleamine derived from L-tryptophan	GRP78↓	Calnexin, ATP sintase β, CHOP, RPB4, β-catenin and SIRT1	-	In Vivo (ob/ob Mice)	↓ Body weight, hepatic steatosis and hepatocyte ballooning	[69]
Docosahexaenoic Acid	Marine fish oil	GRP78↓	CHOP and XBP-1	-	In Vitro (mice hepatocytes) and In Vivo (C57/6J mice)	↓ Lipid accumulation ↓Hepatic Steatosis	[70]
Astragaloside IV	Astragalus membranaceus (Fisch) Extract	GRP78↓	CHOP	AMPK	In Vitro (HepG2) and In Vivo (C57BL/6 mice)	↓ Lipid accumulation and lipogenesis ↓Hepatic steatosis	[75]
Caffeine	-	GRP78↓	Peroxiredoxin 1	-	In Vitro (liver cells isolated from Wistar rats)	↓ Body weight and serum ALT ↓Steatosis, ballooning, inflammatory infiltrate, and fibrosis ↓ Lipid peroxidation and DNA oxidative damage	[71]
Emodin	Hydroxyanthraquinone in the root and rhizome of Rheum palmatum L	GRP78↓	XBP1	SREBP1c and PERK–eIF2α–ATF4	In Vitro (Hepatocytes isolated from liquid fructose-feeding rats)	↓ Bodyweight, liver index, serum TG levels	[72]
Hesperetin	Flavonoid derivative from citrus fruits	GRP78↓	IF2α, JNK, Atf4 and Chop	sXBP1/GRP78	In Vitro (Primary rat hepatocytes and HepG2 cells)	Protects against palmitate-induced cell death in hepatocytes ↓ Palmitate-induced ER stress	[73]
		GRP94↓	ATF6, ATF4, p-PERK, p-IRE1α, IL-1β, IL-6, and TNF-α	-	In Vitro (THP-1 cells)	↓ Hepatic lipid synthesis proteins ↓ERS and inflammation	[74]
NX2-6	Lactobacillus acidophilus extract	GRP78↓	CAT, Nrf-2, ATF6, XBP1, GRP78, p50, and p-ERK	MPKα/Sirt1/PGC-1α/NRF1	In Vitro (HepG2)	↓ Inflammation	[76]
Didemnin B	Marine tunicates	GRP78↓	EEF1A1, GRP78, eIF2α	Inflammatory pathways	In Vitro (HepG2 cells) and In Vivo (male ob/ob mice)	↓ Food consumption and hepatic lipotoxicity in obese mice ↓ Hepatic lipid ↓ Plasma lipid profiles ↑Glucose homeostasis	[77]
Silymarin	Milk thistle extract	GRP78↓	XBP-1	ER stress pathway	In Vitro (mice hepatocytes) and In Vivo (Swiss albino mice)	↓ HDL ↓ERS and inflammation	[10]
Metformin + 1,2,3,4,6-pentagalloyl glucose (PGG)	Antidiabetic + GNMT inducer	HSP72↑	GSTM4, PYC, and RS28	AMPK-related pathways	In Vitro and In Vivo (hepatocytes from C57BL/6J mice)	Reversed weight gain and elevated the level of serum ALT and glucose Induced GNMT expression and decreased c-Myc expression Inhibited hepatic lipid accumulation by reducing lipogenesis via SREBP-1c suppression, improving β-oxidation and inhibiting inflammation	[78]
Empagliflozin	SGLT-2 inhibitors	GRP78↓	Atf4, Elf2α, Chop, Grp78, Grp94, Χbp1, Ire1α, Atf6, mTor, Lc3b, Beclin-1, P62, Bcl-2 and Bax	IRE1a pathway	In Vitro and In Vivo (ApoE -/-mice)	↓ Fasting glucose, total cholesterol, and triglyceride serum levels ↓ ALT and AST ↓ER Stress Markers ↑Hepatic Autophagic Flux ↓ Hepatic Apoptosis	[79]
Exendin-4	GLP-1 Analogs	GRP78↓	CHOP, Beclin, LAMP	-	In Vitro (human hepatocytes) and In Vivo (HFD mice)	↓Fat load in hepatocytes by inducing autophagy.	[80]
Pentoxifylline	Nonselective phosphodiesterase inhibitor	GRP78↓	eIF2α, ATF4, ATF6, p-JNK, and CHOP	TNF-α	In Vitro (cells isolated from rats) and In Vivo (MCD-fed rats)	↓ Serum triglycerides and total cholesterol levels	[81]
N-acetylcysteine	De novo glutathione biosynthesis precursor	HSP60, and HSP70 ↑	ECHS1	-	In Vitro (rat liver cells) and In Vivo (C57BL/6 mice)	↓ Body, liver weight and triglyceride ↓Liver steatosis and apoptosis ↓ERS	[82]

### Natural metabolites

Some natural metabolites have been investigated for their ability to inhibit NAFLD progression through HSPs axis:

#### Lactobacillus acidophilus NX2-6 extract

Lactobacillus acidophilus NX2-6 extract (CFE) emerges as a promising therapeutic agent against NAFLD, offering potential benefits in mitigating the complex pathogenesis of this prevalent liver disorder. Derived from probiotic bacteria, CFE demonstrates efficacy in alleviating hepatic lipid accumulation by promoting triglyceride hydrolysis and fatty acid β-oxidation, as evidenced by increased levels of acetyl-CoA and glycerol in treated cells. Moreover, CFE exerts regulatory effects on key genes and proteins involved in lipogenesis, fatty acid transportation, and lipolysis, resulting in decreased expression of lipid synthesis-related genes and increased expression of lipolytic genes. Additionally, CFE enhances mitochondrial biogenesis and dynamics, improving energy metabolism and ATP production. Furthermore, CFE exhibits antioxidant properties by enhancing the activity of antioxidant enzymes, such as Nrf-2, and reducing endoplasmic reticulum stress, as evidenced by decreased protein expression of ATF6, XBP1, and GRP78. Importantly, CFE also mitigates inflammation by downregulating key genes and proteins involved in the inflammatory cascade, including p50 and p-ERK. Overall, the multifaceted molecular mechanisms of L. acidophilus NX2-6 extract make it a promising candidate for therapeutic intervention in NAFLD, offering potential avenues for future research and clinical application [76].

#### Didemnin B

Didemnin B is a cyclic depsipeptide derived from marine tunicates that selectively inhibits the peptide elongation activity of eukaryotic elongation factor 1 A (EEF1A) by binding to its GTP-bound conformation, thereby preventing protein synthesis. In NAFLD, Didemnin B has been shown to alleviate hepatic lipotoxicity and improve liver pathology in obese mice. Its effects include reducing hepatic lipid content, ameliorating NAFLD histopathology, and decreasing markers of hepatic damage such as plasma ALT and AST levels. Moreover, Didemnin B treatment attenuates ER stress and inflammation in the liver, evidenced by decreased levels of GRP78 and phosphorylated JNK proteins, as well as reduced expression of ER stress and inflammation-related transcripts. These effects are attributed to the inhibition of EEF1A-mediated protein synthesis, suggesting that targeting EEF1A with inhibitors like Didemnin B could be a promising therapeutic strategy for combating ER stress-related metabolic diseases such as NAFLD [77].

### Antidiabetic drugs

#### Metformin

Combining metformin with 1,2,3,4,6-pentagalloyl glucose (PGG) presents a potent therapeutic strategy for NAFLD. Metformin, a well-known medication for type 2 diabetes, and PGG, a potent inducer of glycine N-methyltransferase (GNMT), both individually upregulate GNMT expression, which is downregulated in NAFLD. The combination of metformin and PGG effectively restored GNMT expression in liver tissues of NAFLD mice. This restoration of GNMT expression was accompanied by significant improvements in biochemical markers and histopathological features of NAFLD, including reductions in weight gain, serum transaminases, hepatic steatosis, and inflammation. Notably, the combination therapy also upregulated HSP72, a stress-inducible protective protein that may play a role in preventing hepatic lipid accumulation and improving systemic metabolism. Furthermore, the combination therapy partially rescued the impairment of mitochondrial function induced by metformin alone, suggesting a synergistic action between PGG and metformin in mitigating mitochondrial dysfunction associated with NAFLD. These findings underscore the potential of combined metformin and PGG therapy as a comprehensive approach to address multiple aspects of NAFLD pathogenesis, including GNMT restoration, HSP72 induction, and mitochondrial function, offering a promising avenue for therapeutic intervention in NAFLD management [78].

#### Empagliflozin

Empagliflozin, an SGLT-2 inhibitor, has shown promising effects in attenuating NAFLD progression by targeting multiple pathways involved in its pathogenesis. In a study conducted on ApoE(-/-) mice fed a high-fat diet, five weeks of empagliflozin treatment led to improvements in fasting glucose, total cholesterol, and triglyceride levels, along with reduced markers of liver injury such as serum glutamate-pyruvate transaminase (SGPT). Histological analysis revealed decreased steatosis, intrahepatic ballooning, and lobular inflammation in the empagliflozin-treated group, resulting in a lower NAFLD activity score compared to the control group. Empagliflozin also downregulated the expression of lipogenic enzymes and inflammatory markers while reducing ERS markers. Notably, empagliflozin treatment activated autophagy, as evidenced by increased LC3B expression and decreased mTOR levels, ultimately leading to reduced liver cell apoptosis. Additionally, empagliflozin has been found to decrease the expression of GRP78. This reduction in GRP78 may contribute to the attenuation of liver injury and inflammation observed with empagliflozin treatment in NAFLD [79].

#### GLP-1 analogs(exendin-4)

The potential therapeutic effects of GLP-1 analogs, particularly exendin-4 and liraglutide, on NAFLD through in vitro and in vivo experiments. The researchers demonstrate that GLP-1 analog treatment significantly reduces steatosis (accumulation of fat in hepatocytes) and enhances hepatocyte survivability in primary human hepatocytes loaded with fatty acids. GRP78 plays a significant role in weight loss and improvement, as seen in NAFLD with GLP-1 receptor agonist treatment. GLP-1 analogs like exendin-4 and liraglutide enhance the expression of GRP78 in hepatocytes overloaded with fatty acids. Increased GRP78 levels help mitigate ER stress by properly folding proteins and preventing the accumulation of unfolded proteins. This reduction in ER stress leads to decreased expression of CHOP, a protein that promotes apoptosis under prolonged stress conditions. By alleviating ER stress and reducing apoptosis, GLP-1 receptor agonists promote hepatocyte survival and stimulate autophagy, a process that helps clear excess lipids from liver cells. Consequently, the upregulation of HSPs like GRP78 contributes to the reduction of hepatic steatosis and overall improvement in NAFLD with GLP-1 receptor agonist therapy [80].

### Other drugs

#### Pentoxifylline

Pentoxifylline (PTX) is a nonselective phosphodiesterase inhibitor commonly used in the treatment of vascular diseases due to its ability to improve blood flow and reduce blood viscosity. Recently, it has gained attention for its potential therapeutic effects in NAFLD. PTX acts by reducing the transcription of TNF-α, a cytokine that plays a significant role in inflammation associated with NAFLD. Additionally, PTX has been shown to modulate various steps in the cytokine signaling pathway, indirectly influencing other inflammation and stress-related markers. Studies, including those using animal models, have demonstrated that PTX can mitigate the hepatic steatosis and inflammation typical of NAFLD, primarily by decreasing cytokine-mediated inflammation and alleviating ER stress. Models of NAFLD induced by an MCD diet have shown that PTX can attenuate the increase in GRP78 levels that is typically observed under such stress conditions. This suggests that PTX can reduce ER stress, part of which involves moderating the upregulation of GRP78 [81].

#### N-acetylcysteine (NAC)

N-acetylcysteine (NAC) is an antioxidant that serves as a precursor to glutathione, a crucial molecule in detoxification and antioxidant defense within cells. Research has shown that long-term use of NAC can be beneficial in reducing the progression NAFLD. Specifically, NAC appears to ameliorate the harmful effects of a high-fat diet on the liver by enhancing the expression of heat shock proteins (HSPs), such as HSP70 and HSP60. These proteins play vital roles in protein folding and protecting cells from stress-induced damage. By upregulating HSPs, NAC helps mitigate ER stress and activates the UPR, essential mechanisms for maintaining cellular health in the liver under stress conditions associated with fat accumulation. Thus, NAC’s ability to enhance antioxidant defenses and improve protein homeostasis in hepatocytes makes it a promising agent in managing and potentially reversing NAFLD [82].

### Diet effects

#### Isocaloric time-restricted feeding

Isocaloric Time-Restricted Feeding (TRF) is a dietary strategy where the total daily caloric intake remains constant, but the consumption of calories is restricted to a specific time window each day. This approach does not necessarily aim to reduce calorie intake but to confine eating to certain hours, typically aligning with the body’s circadian rhythms. In the context of NAFLD, short-term isocaloric TRF has shown potential in reducing hepatic inflammation, a key factor in the progression of NAFLD, despite not impacting overall weight loss or liver steatosis significantly. The mechanism behind this benefit may involve the modulation of ER stress response. ER stress is closely linked to metabolic dysregulation, and by potentially minimizing this stress, TRF could improve the liver’s metabolic functions and reduce the activation of inflammatory pathways, thereby helping to prevent the progression of NAFLD. This approach suggests that altering meal timing without changing caloric content might provide a therapeutic advantage by aligning food intake with the body’s natural biological rhythms, thereby enhancing metabolic health. TRF was shown to reduce the expression of GRP78, suggesting a reduction in ER stress within the liver cells [83].

#### 3021 Meal replacement powder

The 3021 meal replacement powder (MRP) is a dietary supplement composed of various plant-based ingredients like cereals, beans, tubers, and additional plant parts such as soybean dietary fiber, collagen, and soy protein. It is designed to regulate glucolipid metabolism, enhance satiety, and reduce caloric intake, thereby facilitating weight loss and potentially mitigating obesity-related diseases such as NAFLD. According to research findings, the MRP can effectively reduce lipid accumulation in hepatocytes and mitigate ERS markers such as GRP78 and GRP94. By suppressing the expression of these proteins, which are crucial in the UPR, the MRP can prevent the misfolding of proteins that exacerbates liver inflammation and damage under stress conditions, thereby protecting against the progression of NAFLD [84].

#### Exercise

Aerobic exercise refers to a form of physical activity characterized by sustained, rhythmic movements that increase heart rate and breathing for an extended period. It typically involves activities such as running, swimming, cycling, or brisk walking, which engage large muscle groups and require continuous oxygen consumption to generate energy.mice subjected to high-fat diet-induced NAFLD exhibited elevated levels of GRP78 and ATF6 proteins and their corresponding mRNA levels compared to mice on a standard diet. However, aerobic exercise intervention effectively reversed these diet-induced changes, leading to lower levels of GRP78 and ATF6 expressions in the liver. This downregulation of GRP78 and ATF6 expressions correlated with improvements in various NAFLD-related parameters, including reduced body weight, liver weight, liver index, histopathological abnormalities, and serum lipid profiles. These findings suggest that aerobic exercise mitigates ER stress in the liver of mice with NAFLD, potentially contributing to the amelioration of the disease [85, 86].

### Gastric bypass surgery

Roux-en-Y gastric bypass (RYGB) surgery is a type of bariatric surgery that alters the gastrointestinal tract to reduce food intake and nutrient absorption, thereby promoting significant weight loss. RYGB has been shown to offer protective effects against NAFLD and endoplasmic reticulum stress (ERS). The surgery appears to mitigate NAFLD by reducing hepatic triglyceride accumulation, steatosis, and inflammation, likely through mechanisms that extend beyond just weight loss. RYGB also reduces ERS markers in the liver, including the key sensors and downstream effectors such as IRE1, PERK, and ATF6. Additionally, the surgery reduced the expression of GRP78, a chaperone involved in protein folding and ERS response, indicating that RYGB can ameliorate the cellular stress associated with obesity and liver disease. These effects may also be mediated by changes in gut hormone secretion, such as increased levels of GLP-1 post-surgery, enhancing metabolic improvements and contributing to the overall protective effects of RYGB against metabolic and hepatic complications [87].

## Conclusion and remarks

This study summarized the role of HSPs in the pathogenesis and treatment of NAFLD. In NAFLD, HSPs play crucial roles in mitigating liver damage and disease progression. The novelty of this review lies in its comprehensive exploration of the multifaceted roles that HSPs play in the pathogenesis and therapeutic potential within NAFLD. This review distinguishes itself by systematically categorizing different HSP families, such as HSP20, HSP27, HSP60, HSP70, and HSP90, and highlighting their distinct mechanisms in modulating lipid metabolism, inflammation, oxidative stress, and autophagy, core processes in NAFLD development. The review offers an in-depth molecular understanding, particularly emphasizing how HSPs can either exacerbate or mitigate disease progression based on their expression levels and interactions with cellular stress responses like ER stress. A key novelty lies in the identification of HSPs as both biomarkers for disease severity and as potential therapeutic targets. The review’s focus on the therapeutic implications of modulating HSPs, including natural compounds like phytochemicals and antioxidants that can influence HSP expression, provides a fresh perspective on possible treatment strategies. By weaving together molecular, therapeutic, and clinical aspects, the review pushes the frontier of how HSPs can be integrated into a more holistic treatment framework for NAFLD.These proteins exhibit anti-inflammatory effects by inhibiting inflammatory pathways and reducing pro-inflammatory cytokine production. Additionally, they protect liver cells from apoptosis induced by oxidative stress and inflammation, thereby preserving liver function. HSPs also regulate lipid metabolism by interacting with key enzymes and transcription factors involved in lipid synthesis and storage, influencing the accumulation of fat in the liver. Moreover, HSPs have antioxidant properties, scavenging reactive oxygen species and protecting cells from oxidative damage. Collectively, HSPs serve as important protective factors in NAFLD, highlighting their potential as therapeutic targets for the disease. Small HSPs regulate disease progression by influencing autophagy. In NAFLD, HSP20 exacerbates lipotoxicity by inhibiting autophagy, while HSP27, when phosphorylated, promotes autophagy, aiding hepatic lipid clearance. Additionally, androgen signaling through the AR regulates HSP27 expression, further implicating HSPs in NAFLD pathogenesis. HSP60 in NAFLD primarily regulates lipid metabolism, enhances glucose tolerance and insulin sensitivity, suppresses inflammation, and modulates mitochondrial biogenesis. It is considered a potential therapeutic target for NAFLD due to its role in regulating lipid metabolism, inflammation, and mitochondrial function. HSP70, on the other hand, plays a complex role in NAFLD, where it promotes hepatic steatosis by stimulating lipogenesis but also exhibits anti-inflammatory properties by inhibiting NF-κB activation and JNKs. Its upregulation in obese individuals suggests involvement in metabolic disturbances, making it a potential therapeutic target for managing NAFLD and related metabolic disorders. The comprehensive exploration of GRP78 and its regulatory mechanisms in NAFLD reveals its pivotal role in disease progression. GRP78, a marker for ERS, exhibits upregulation in NAFLD, particularly in advanced stages like NASH, correlating with heightened inflammation, hepatocyte apoptosis, and macrophage infiltration. Notably, various regulators, including HIF-1α, eEF1A-1, PDIA3, and others, intricately modulate GRP78 expression, impacting ER stress and lipotoxicity pathways. These regulators influence NAFLD progression through diverse mechanisms, such as inflammation, oxidative stress, and mitochondrial dysfunction. The findings underscore the complex interplay between ER stress, lipogenesis, and inflammation in NAFLD pathogenesis, positioning GRP78 as a crucial mediator and potential therapeutic target for mitigating disease progression and associated complications. HSP72 induction improves glucose tolerance, reduces hepatic insulin resistance, and prevents lipid accumulation in the liver while protecting against hepatocellular death and oxidative stress. Similarly, HSP90, particularly HSP90β, contributes to dysregulated lipid homeostasis by stabilizing transcription factors involved in lipid synthesis and promoting PPARγ activity, exacerbating hepatic steatosis. Inhibition of HSP90 shows promise in mitigating lipid dysregulation and hepatic steatosis. Furthermore, HSP90 interacts with USP14 to stabilize enzymes involved in NAFLD progression and with albumin to maintain mitochondrial function and protect against oxidative stress. Thus, targeting these HSPs represents a promising avenue for managing NAFLD and its associated metabolic complications. Therapeutic interventions targeting HSPs in NAFLD can be classified into several categories, including phytochemicals, traditional Chinese medicine formulations (e.g., Hugan Qingzhi), and specific compounds like lipoic acid, abietic acid, and melatonin. These interventions exert their effects through diverse mechanisms, such as modulation of endoplasmic reticulum stress, regulation of lipid metabolism, and attenuation of oxidative stress and inflammation. Therapeutic strategies targeting HSPs in NAFLD commonly involve the modulation of molecular mechanisms aimed at alleviating cellular stress and restoring metabolic homeostasis. HSPs, notably HSP70 and HSP90, play pivotal roles in protein folding, cellular stability, and stress response pathways. Pharmacological interventions and natural compounds targeting HSPs aim to mitigate ER stress, oxidative stress, and inflammation, key contributors to NAFLD progression. By enhancing protein folding and refolding, HSP-targeted therapies restore proper protein function, alleviate mitochondrial dysfunction, and reduce inflammatory responses in hepatic cells. Additionally, HSP modulation influences lipid metabolism pathways, including de novo lipogenesis and fatty acid oxidation, thus aiding in the prevention and management of hepatic lipid accumulation. These interventions offer multifaceted effects, providing potential avenues for the development of effective treatments for NAFLD by targeting central molecular mechanisms involved in disease pathogenesis. Resmetirom (Rezdiffra) is an investigational drug designed as a selective thyroid hormone receptor beta (THR-β) agonist to reduce liver fat, inflammation, and fibrosis in NAFLD. In March 2024, the United States granted Food and Drug Administration (FDA)-approval for resmetirom to be used alongside diet and exercise in treating adults with noncirrhotic non-alcoholic steatohepatitis who have moderate to advanced liver fibrosis, specifically fibrosis stages F2 to F3. Evaluating its effects on HSPs is important because HSPs are molecular chaperones involved in protein folding and cellular stress responses, which are critical in liver physiology and pathology. Understanding how resmetirom interacts with HSPs can provide insights into its mechanism of action, potentially enhance its therapeutic efficacy, identify biomarkers for treatment response, uncover resistance mechanisms, and contribute to personalized treatment strategies, ultimately improving outcomes for patients with NAFLD. The primary weakness is the lack of comprehensive clinical studies in the field, which limits the review’s ability to make definitive conclusions about the therapeutic potential of targeting HSPs in NAFLD. Much of the evidence is based on preclinical studies, such as in vitro experiments and animal models, which do not always translate well into clinical outcomes in human populations. This gap in clinical evidence is a significant hurdle for the practical application of HSP modulation as a therapeutic strategy for NAFLD. Additionally, many of the original studies focus on specific HSPs, leaving a broader understanding of how these proteins interact with each other and other cellular mechanisms in NAFLD underexplored.

Furthermore, while our review thoroughly discusses the molecular mechanisms by which HSPs influence NAFLD progression, the field lacks large-scale clinical trials to confirm whether manipulating these pathways will have consistent therapeutic effects in human patients. The absence of these clinical studies makes it difficult to gauge the safety, efficacy, and long-term outcomes of HSP-targeted treatments. Therefore, the limitations are primarily due to the nascent stage of research in this area and the dependence on preclinical models, not the review itself. Finally, while it briefly touches on phytochemicals, the review could have expanded more on novel, under-explored compounds and their precise modes of action in relation to HSP modulation, which would have added further value to the therapeutic discussion.

## Data Availability

No datasets were generated or analysed during the current study.

## Declarations

### Generative AI and AI-assisted technologies in the writing process

During the preparation of this work, the authors used ChatGPT by OpenAI to improve paper readability. After using this tool/service, the authors reviewed and edited the content as needed and took full responsibility for the publication’s content.
